# Hypothyroidism impairs skeletal muscle regeneration after injury by altering myogenic and nonmyogenic pathways

**DOI:** 10.1172/jci.insight.197761

**Published:** 2026-03-23

**Authors:** Paola Aguiari, Valentina Villani, Yan-Yun Liu, Gianni Carraro, Gregory A. Brent, Laura Perin, Anna Milanesi

**Affiliations:** 1 GOFARR Laboratory, Children’s Hospital Los Angeles, Division of Urology, Saban Research Institute, Los Angeles, California, USA.; 2 Department of Medicine, David Geffen School of Medicine at UCLA–VA Greater Los Angeles Healthcare System, Los Angeles, California, USA.; 3 Children’s Hospital Los Angeles, Division of Pediatric Surgery, Saban Research Institute, Los Angeles, California, USA.; 4 Keck School of Medicine, University of Southern California, Los Angeles, California, USA.

**Keywords:** Cell biology, Endocrinology, Muscle biology, Muscle

## Abstract

Thyroid hormone signaling is an essential regulator of skeletal muscle development, function, and metabolism, yet the specific signaling pathways required for muscle regeneration are not yet defined. We used scRNA-seq and the FUCCI (fluorescent ubiquitination-based cell cycle indicator) reporter mouse model to examine how hypothyroidism impacts repair processes after cardiotoxin-induced injury in mice. During regeneration, and up to 2 months after injury, hypothyroid muscles displayed smaller myofibers and a shift to slower oxidative fiber types. scRNA-seq of tibialis anterior muscle during regeneration revealed that hypothyroidism reduced myogenic-lineage diversity. Cell cycle analysis confirmed delayed cell cycle progression at 5 and 14 days after injury, with skeletal muscle stem cells stalled at the G_1_/S transition, hindering differentiation. Transcriptomic data revealed altered nonmyogenic dynamics, including elevated activated fibro-adipogenic progenitors (FAPs) early in repair and persistent proinflammatory macrophages. Integrative regulon and ligand-receptor analysis further demonstrated that triiodothyronine acted through dual modes: a direct transcriptional control of myogenic cell cycle and oxidative programs and an indirect paracrine remodeling mediated by FAP and immune signaling networks. This study identified what we believe to be novel effects of hypothyroidism on myogenic heterogeneity and impaired tissue repair, offering insights into muscle-wasting mechanisms relevant to hypothyroidism-associated myopathy and sarcopenia.

## Abstract 2

Hypothyroidism impairs muscle regeneration by disrupting cell cycle progression, reducing myogenic diversity, and by altering fibro-adipogenic and immune cell dynamics.

## Introduction

Skeletal muscle regeneration is a highly regulated process driven by the orchestrated interplay of resident muscle stem cells (MuSCs), immune cells, and fibro-adipogenic progenitors (FAPs) to repair tissue damage following injury (1). This process hinges on MuSC activation, proliferation, and differentiation, and it is regulated by intrinsic myogenic programs and extrinsic signals, including growth factors, cytokines, and hormones (2, 3). Among these, thyroid hormones, particularly triiodothyronine (T3), are pivotal in skeletal muscle development, homeostasis, and repair, influencing metabolic activity, mitochondrial function, and myogenic gene expression (4, 5). T3 directly controls MuSC activation by inducing *Myod1* expression to initiate cell cycle entry and later promotes cell cycle exit and differentiation through myogenin and myosin heavy chain isoform expression (5, 6).

The genomic actions of T3 are mediated by thyroid hormone nuclear receptors (THRs), which act as ligand-inducible transcription factors (7). Disruption of thyroid hormone signaling — as seen in mouse models such as D2/D3 knockouts (deiodinase 2/deiodinase 3; predominant thyroid hormone–activating and –deactivating enzymes, respectively) or thyroid hormone receptor α (THRA) mutations — impairs regeneration, leading to defective myogenesis and premature sarcopenia (8–11). In addition, hypothyroid patients commonly report symptoms of hypothyroid myopathy, like exercise intolerance, myalgias, cramps, stiffness, and myoedema (12). Despite these insights, the cellular and molecular mechanisms by which hypothyroidism alters muscle repair remain poorly understood. In particular, how thyroid hormone deficiency alters myogenic lineage commitment, cell cycle dynamics, and interactions between MuSCs and supporting cell types has yet to be fully elucidated.

Recent advances in scRNA-seq have revolutionized our ability to dissect cellular heterogeneity and transcriptional profile changes during muscle regeneration (13–16). However, capturing the real-time cell cycle changes of MuSCs during regeneration has remained challenging.

In this study, we combined scRNA-seq with a fluorescent ubiquitination-based cell cycle indicator (FUCCI) reporter mouse model to investigate how hypothyroidism affects skeletal muscle regeneration after cardiotoxin-induced injury. Our findings reveal that hypothyroidism reduces cellular diversity within the myogenic lineage, increases the proportion of activated FAPs early in regeneration, and alters immune cell composition throughout the repair process, revealing previously uncharacterized effects of hypothyroidism on muscle repair. These findings offer more insights into the mechanisms underlying hypothyroid myopathy and identify thyroid hormone signaling as a potential therapeutic target for improving muscle repair.

## Results

### Skeletal muscle regeneration after injury is impaired during hypothyroidism.

**Figure 1:**
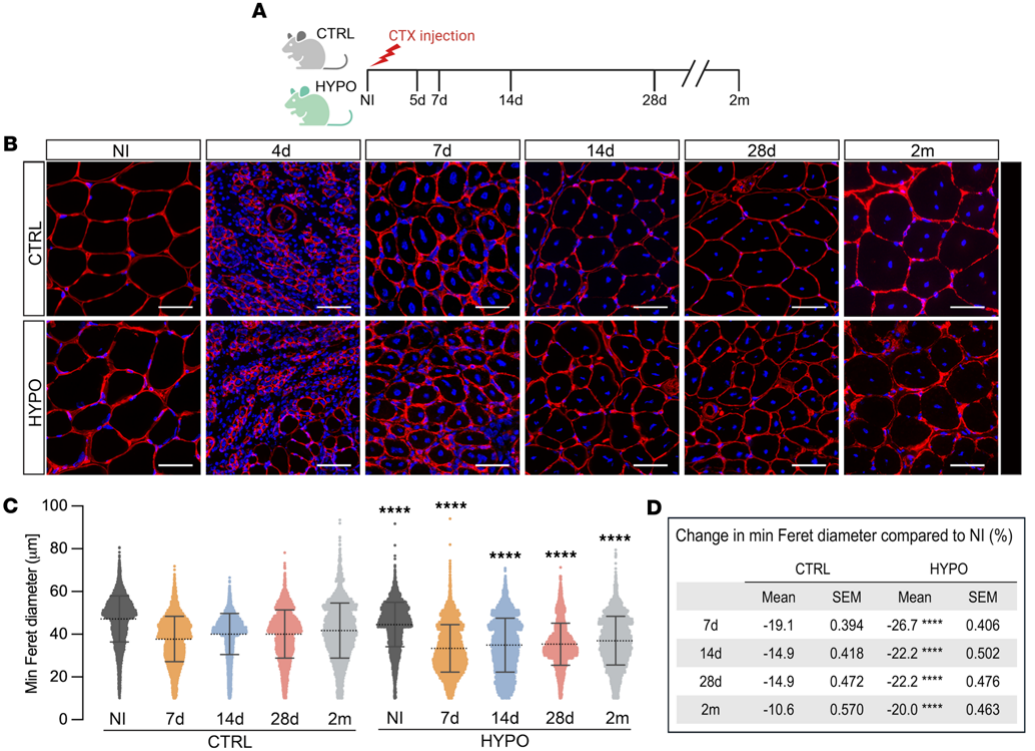
Skeletal muscle regeneration after injury: hypothyroid versus control. (**A**) Experimental design overview. Tibialis anterior muscles (TAMs) were collected from control and hypothyroid mice before injury and 4, 7, 14, and 28 days and 2 months after cardiotoxin-induced injury. Created in BioRender (Aguiari P, 2026, https://BioRender.com/9ywq1dw). (**B**) Immunofluorescence of TAM sections from control and hypothyroid mice before and 7, 14, and 28 days and 2 months after cardiotoxin-induced injury. Red, laminin; blue, nuclei (DAPI). Scale bars: 50 μm. (**C**) Scatter dot plot of minimum Feret diameter of TAM fibers from control and hypothyroid mice before and 7, 14, and 28 days and 2 months after injury. (**D**) Table showing the percentage decrease of minimum Feret diameter of regenerating skeletal muscle fibers in control and hypothyroid mice relative to the uninjured muscle. Data are presented as mean ± SEM. *n* = 3. One-way ANOVA and paired 2-tailed Student’s *t* tests were used for statistical analysis. ****P < 0.001, in hypothyroid compared with time point–matched control fibers. The experiment was repeated 3 times independently under identical conditions. CTRL, control; HYPO, hypothyroid; CTX, cardiotoxin; NI, before injury; d, days after injury; m, months after injury.

To assess the impact of thyroid hormone deficiency on muscle regeneration, we induced injury with cardiotoxin (CTX) in the tibialis anterior muscle (TAM) of hypothyroid mice, compared with euthyroid control mice, and monitored recovery over 2 months (Figure 1A). Hypothyroidism was induced in mice 3 weeks before injury and maintained for the duration of the study. We confirmed that the dietary/propylthiouracil regimen produced systemic hypothyroidism before muscle injury, by measuring circulating T3 levels at 3 key time points (Supplemental Figure 1).

Uninjured hypothyroid muscle fibers were significantly smaller than those of control mice, as measured by minimal Feret diameter (Figure 1, B and C). At 4 days after injury (dpi), both groups exhibited similar histological appearance, with no major morphological differences. However, starting at day 7, the hypothyroid group displayed a marked and sustained reduction in myofiber size compared with controls. This included significantly smaller cross-sectional areas and minimal Feret diameters across all subsequent time points (Figure 1, B and C). Notably, the degree of myofiber size loss after injury, calculated as the percentage decrease relative to uninjured muscle, was significantly greater in hypothyroid mice, as shown in Figure 1D.

These findings indicate that hypothyroidism delays muscle fiber growth during regeneration and exacerbates muscle fiber atrophy following injury. The persistence of smaller fiber size up to 2 months after injury suggests incomplete recovery and impaired regenerative capacity in the hypothyroid group.

### scRNA-seq reveals altered muscle-resident cell dynamics in hypothyroid mice.

**Figure 2:**
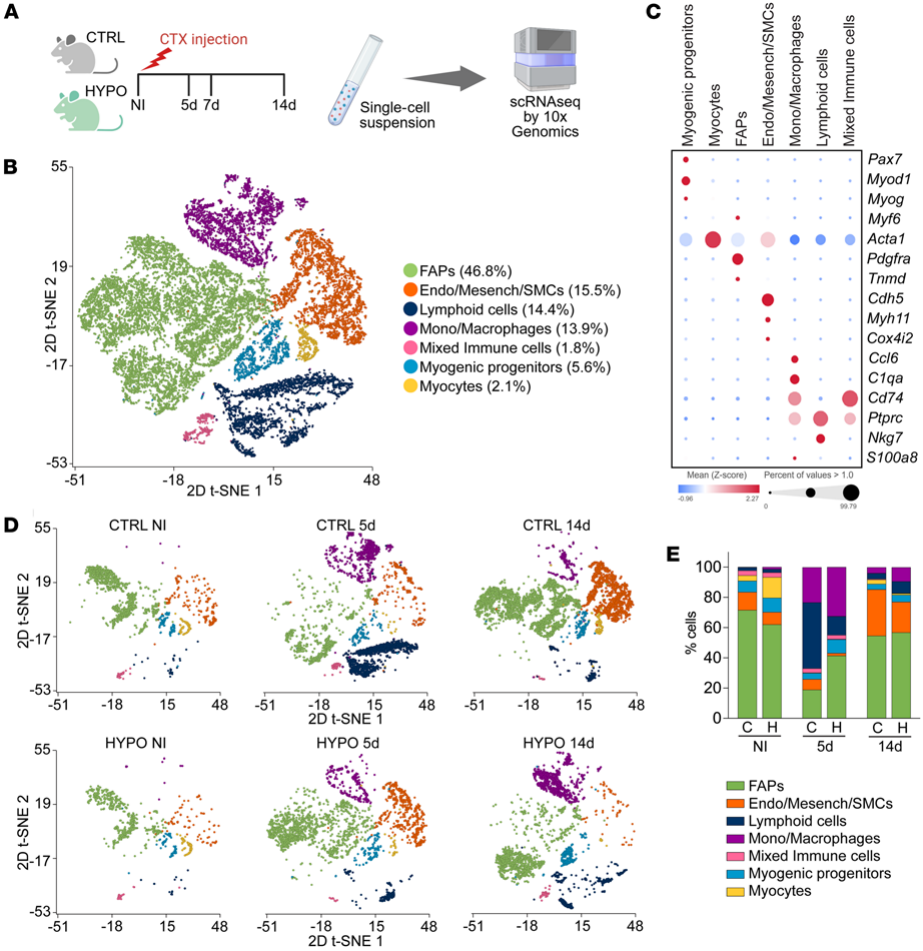
scRNA-seq of TAM shows different muscle-resident cell dynamics in response to injury in hypothyroid versus control mice. (**A**) Experimental design overview. Single cells were collected from TAMs isolated from control and hypothyroid mice before injury and 5 and 14 days after cardiotoxin-induced injury, and subjected to scRNA-seq followed by downstream analysis. Created in BioRender (Aguiari P, 2026, https://BioRender.com/rz1yprc). (**B**) t-SNE embedding of integrated dataset from control and hypothyroid muscle scRNA-seq datasets from all the time points, colored by population. (**C**) Bubble plot showing the expression of canonical markers in each population. Average gene expression values are scaled. (**D**) t-SNE embedding of integrated dataset from control and hypothyroid scRNA-seq data colored by population and grouped by time point. (**E**) Graph showing the relative proportion of each cell type in control and hypothyroid regenerating muscles at the different time points, shown as a percentage. Number of cells for each sample: CTRL NI, 2,303; CTRL 5d, 4,852; CTRL 14d, 6,588; HYPO NI, 1,055; HYPO 5d, 4,312; HYPO 14d, 3,299.

To investigate the molecular effects of hypothyroidism on skeletal muscle regeneration, we performed scRNA-seq on TAM from control and hypothyroid mice at 0, 5, and 14 dpi, capturing MuSC proliferation (5 days) and differentiation/remodeling (14 days) phases (Figure 2A). After quality control, the scRNA-seq datasets contained an average of 3,735 ± 1,786 cells per sample, and the integrated dataset included a total of 22,409 cells. Unsupervised graph-based clustering of the integrated data identified 9 distinct clusters (Supplemental Figure 2, A and B, and Supplemental Dataset 1). Based on differential expression of canonical markers (13, 15) and by combining the FAP clusters into one single cluster, we reclassified these 9 clusters into 7 broad groups of known muscle-resident cell types, shown by 2D t-distributed stochastic neighbor embedding (t-SNE) (Figure 2, B and C, and Supplemental Dataset 2). FAPs represented the dominant cluster (46.8%), followed by endothelial/mesenchymal cells (15.5%), lymphoid cells (B, T, and NK cells) (14.4%), myeloid cells (monocytes and macrophages) (13.9%), a cluster of mixed immune cells (1.8%), myogenic progenitors (5.6%, cells expressing *Pax7*, *Myod1*, or *Myog*), and a small population of mature myocytes (6%, expressing *Myh1* and *Acta1*, a marker of terminally differentiated cells). Hypothyroid muscles showed distinct cell dynamics: uninjured samples had a 3.8-fold increase in myocytes, while at 5 dpi, FAPs and myogenic progenitors increased 2.2- and 2.3-fold, respectively, compared with controls. By 14 days, hypothyroid samples exhibited a 2.0-fold increase in lymphoid cells and a 2.4-fold increase in monocytes and macrophages, and a 2.9-fold drop in myocytes (Figure 2, D and E), suggesting altered cellular coordination during repair.

### Hypothyroidism reduces myogenic-lineage heterogeneity.

To examine how hypothyroidism influences transcriptional programs during muscle regeneration, we focused on myogenic-lineage cells from integrated control and hypothyroid datasets across regeneration stages. We selected the myogenic progenitor and myocyte clusters, and we initially identified 1,711 cells. After exclusion of 2 subpopulations enriched in glial cell, Schwann cell, and erythrocyte markers (Supplemental Figure 2B, Supplemental Figure 3, and Supplemental Dataset 3), the final analysis included 1,463 myogenic cells.

**Figure 3:**
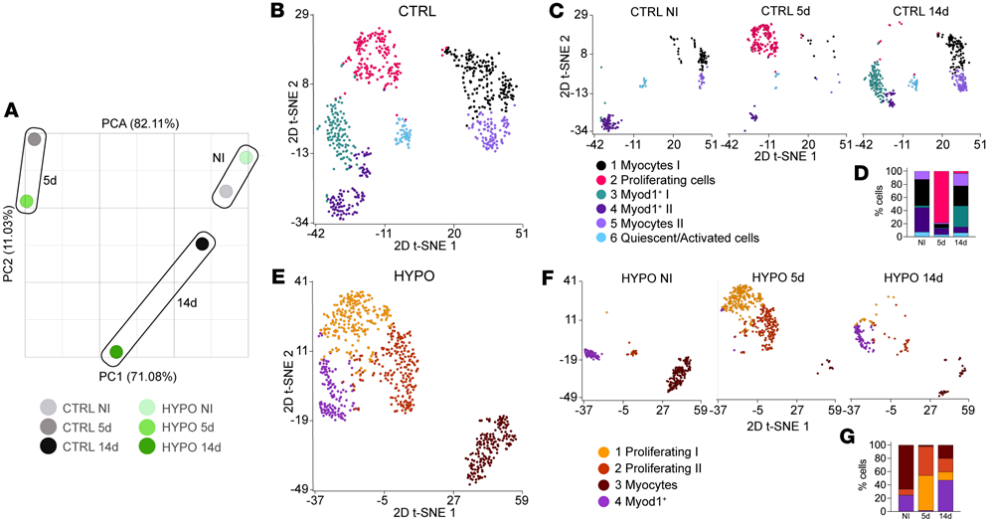
Hypothyroidism alters transcriptional profiles, cellular composition, and regenerative gene programs in the myogenic lineage during muscle regeneration. (**A**) 2D principal component analysis (PCA) plot for control and hypothyroid myogenic-lineage cells at the different time points before injury and 5 and 14 dpi. (**B**) t-SNE visualization of control myogenic scRNA-seq data, with color-coded clusters. (**C**) t-SNE visualization of control myogenic scRNA-seq data, color-coded by cluster and grouped by time point. (**D**) Graph depicting the relative proportions of each cluster in control myogenic-lineage cells across different time points shown as percentage. Number of cells for each sample: CTRL NI, 163; CTRL 5d, 201; CTRL 14d, 344. (**E**) t-SNE visualization of hypothyroid myogenic-lineage scRNA-seq data colored by cluster. (**F**) t-SNE visualization of hypothyroid myogenic-lineage scRNA-seq data colored by cluster and grouped by time points. (**G**) Graph showing the relative proportion of each cluster in hypothyroid myogenic-lineage cells at different time points. Number of cells for each sample: HYPO NI, 208; HYPO 5d, 399; HYPO 14d, 148. CTRL, control; HYPO, hypothyroid.

Pseudo-bulk principal component analysis (PCA) captured global transcriptional trends. Principal component 1 (PC1), explaining 71.08% of the variance, separated noninjured (NI) samples from samples 5 dpi. At 14 dpi, control samples clustered closely with NI, suggesting recovery, while hypothyroid samples diverged along both PC1 and PC2, indicating persistent transcriptional alterations (Figure 3A).

To explore temporal and condition-specific changes, we analyzed hypothyroid and control myogenic groups separately. This yielded 708 cells from control and 755 from hypothyroid samples. In control muscles, unsupervised clustering identified 6 distinct clusters that we classified based on expression of canonical myogenic and cell cycle markers (Supplemental Figure 4A and Supplemental Dataset 4): quiescent/activated progenitors (expressing *Pax7*, *Myf5*, *Ccnd1*, and *Cdk6*), present across all time points; proliferating myoblasts (expressing *Ccne1*, *Cdk2*, *Ccna2*, *Cdk1*, and *Myog*), peaking at 5 dpi; two *Myod1^+^* progenitor clusters, *Myod1*^+^ I (expressing *Pax7*, *Myf5*, *Ccne1*, and *Ccne2*, enriched in NI muscles) and *Myod1^+^* II (expressing *Myh6*, *Myl1*, *Acta1*, and *Myh4*, enriched at 14 days), representing cells poised for differentiation; and two differentiated myocyte clusters (Myocytes I and II), expressing *Myh1* and *Myh4*, respectively, both present at baseline and 14 dpi (Figure 3, B–D).

Based on Gene Ontology (GO) analyses of differentially expressed genes, Myocytes I displayed enrichment for pathways that include mTOR signaling, mitochondrial translation, tricarboxylic acid cycle, and respiratory electron transport chain, strongly suggesting that these cells adopt a transcriptional program characteristic of oxidative, metabolically active muscle fibers (17, 18). In contrast, Myocytes II exhibited enrichment for GO pathways such as Rho GTPase signaling, cell cycle progression, translation initiation complex formation, HSF1 activation, and VEGF-VEGFR pathway (Supplemental Figure 5 and Supplemental Dataset 5).

Unsupervised graph-based clustering of hypothyroid myogenic cells identified only 4 clusters (Figure 3E, Supplemental Figure 4B, and Supplemental Dataset 6), indicating reduced heterogeneity. These included a single *Myod1^+^* population spanning NI and 14 dpi; two proliferating clusters (Proliferating I and II), both expressing cell cycle (*Ccne1*, *Cdk1*, *Ccna2*, *Ccnb1*) and cell cycle exit genes (*Myog*, *Ccnd3*, *Cdkn1c*) (Figure 3, F and G), but differing in their transcriptional profiles: Proliferating I enriched for mTOR signaling, ribosomal biogenesis, and stress response, and Proliferating II showing markers of mitochondrial biogenesis, extracellular matrix (ECM) remodeling, autophagy, and cytoskeletal reorganization (Supplemental Figure 6); and one differentiated myocyte cluster (expressing *Myh1* and *Myh4*).

The analyses did not identify a distinct cluster for quiescent/activated cells in the hypothyroid dataset. However, proliferating cells (Proliferating II) expressing Notch signaling in the uninjured hypothyroid muscle could represent activated myogenic cells. Proliferative cells were detected even in uninjured hypothyroid muscle, with a moderate increase at 5 days and a marked 10-fold rise by 14 dpi (Figure 3G). Differentiated myocytes were significantly reduced at both 5 days and 14 days in hypothyroid regenerating muscles when compared with controls (Supplemental Table 1).

GO term analysis comparing control with hypothyroid myogenic cells 5 days after muscle injury revealed enrichment of pathways related to muscle cell differentiation, activation, adaptation, and skeletal muscle contraction (Supplemental Figure 7 and Supplemental Dataset 7). By 14 dpi, control myogenic cells showed increased activation of MET-PTK2 signaling, integrin-mediated cell surface interactions, nonintegrin membrane-ECM interactions, and collagen biosynthesis (Supplemental Figure 7 and Supplemental Dataset 8). These findings suggest that hypothyroidism disrupts normal regenerative progression by reducing cellular diversity within the myogenic lineage, delaying the transition from proliferation to differentiation, and impairing the reestablishment of mature muscle fiber populations. Hypothyroid muscles showed abnormal transcriptional programs and ongoing proliferation, which might likely hinder muscle repair and function after injury.

### Hypothyroidism alters proliferation and differentiation in the myogenic lineage.

**Figure 4:**
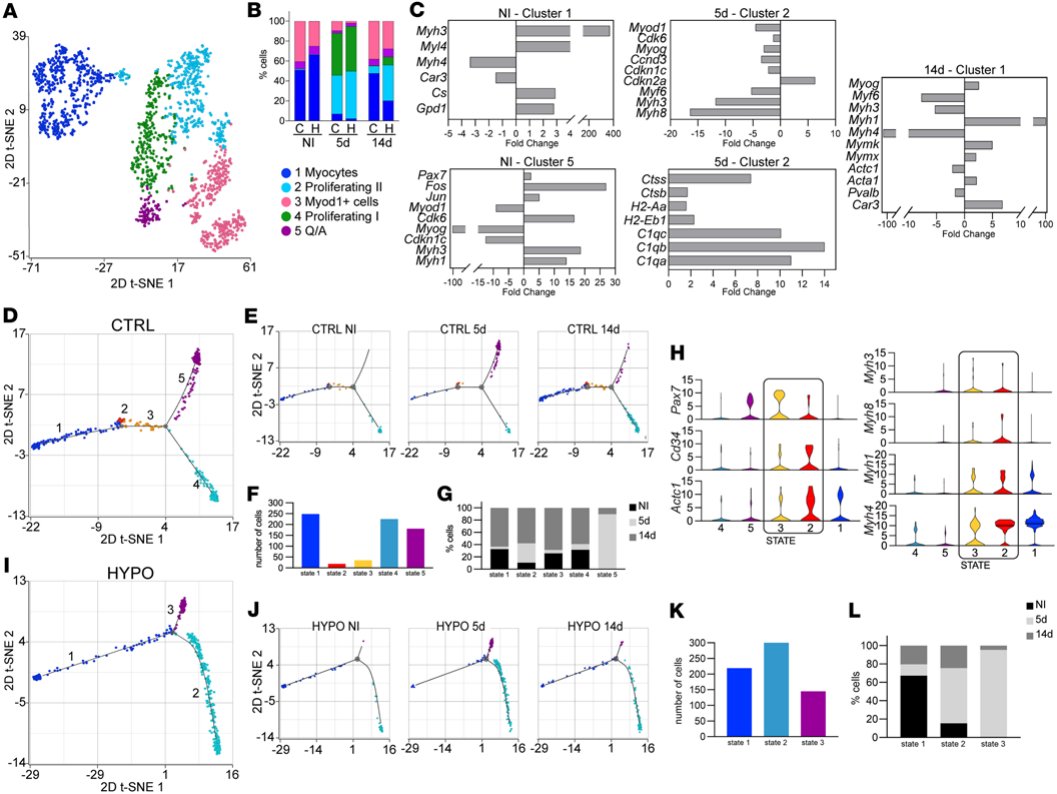
scRNA-seq and trajectory analysis reveal altered myogenic cell state transitions and impaired progression in hypothyroid muscle regeneration. (**A**) t-SNE visualization of the integrated scRNA-seq datasets from control and hypothyroid myogenic-lineage cells, with clusters distinguished by color. (**B**) Graph depicting relative proportions of clusters in each sample shown as percentage. Number of cells for each sample: CTRL NI, 163; CTRL 5d, 201; CTRL 14d, 344; HYPO NI, 208; HYPO 5d, 399; HYPO 14d, 148. (**C**) Table showing fold change in gene expression of selected genes for cells in Myocytes (cluster 1), Quiescent/Activated (cluster 5), and Proliferating II (cluster 2) clusters, in uninjured muscle and 5 days and 14 dpi, comparing hypothyroid and control myogenic-lineage cells. (**D**) Monocle trajectory plots for control datasets showing the distribution of myogenic cells in 5 states. (**E**) Monocle trajectory plots split by time point, in uninjured muscle and 5 days and 14 dpi, showing the distribution of control myogenic cells in 5 states. (**F**) Graph showing the number of cells in each control Monocle trajectory state. (**G**) Graph showing the percentage of cells from each time point for each control Monocle trajectory state. (**H**) Violin plots showing gene expression for selected genes in the control-specific myocyte states. (**I**) Monocle trajectory plots for hypothyroid datasets showing the distribution of hypothyroid myogenic cells in 3 states. (**J**) Monocle trajectory plots split by time point, in uninjured muscle and 5 days and 14 dpi, showing the distribution of hypothyroid myogenic cells in 3 states. (**K**) Graph showing the number of cells in each hypothyroid Monocle trajectory state. (**L**) Graph showing the percentage of cells from each time point for each hypothyroid Monocle trajectory state. C, control; H, hypothyroid.

To investigate the effects of hypothyroidism on myoblast proliferation and differentiation, we analyzed the integrated myogenic-lineage data from control and hypothyroid groups and applied graph-based clustering. We identified 5 distinct clusters (Figure 4, A and B, and Supplemental Dataset 3) based on differential gene expression of key markers of myogenesis and cell cycle regulation (Supplemental Figures 8 and 9). The identified populations aligned closely with separate control and hypothyroid analyses, comprising quiescent/activated cells (Q/A, cluster 5), *Myod1^+^* cells (cluster 3), differentiated myocytes (cluster 1), and 2 distinct clusters of proliferating cells: Proliferating I (cluster 4), representing actively cycling myoblasts with upregulated cell cycle genes (*Ccne1*, *Cdk2*, *Ccna2*, *Cdk1*, *Ccnb1*); and Proliferating II (cluster 2), exhibiting increased *Myod1* and *Myog* expression, indicative of cells transitioning to exit the cell cycle toward differentiation (Figure 4B).

Comparative gene analysis between hypothyroid and control samples identified transcriptional differences across quiescent/activated cells, proliferating myoblasts, and differentiated myocytes (Figure 4C and Supplemental Dataset 9). In uninjured muscle (NI), quiescent/activated cells (cluster 5) from hypothyroid samples exhibited a pre-activated state, characterized by upregulation of activation markers *Fos* and *Jun* as well as myosin genes (*Myh1*, *Myh3*) (Figure 4C). This was accompanied by the downregulation of the thyroid hormone target genes *Myod1* and *Myog*, and the cell cycle inhibitor p57 (*Cdkn1c*), compared with their euthyroid counterparts (Figure 4C).

Hypothyroid Proliferating II cells (cells poised for differentiation, cluster 2) at 5 dpi exhibited downregulation of *Myod1*, genes involved in cell cycle exit (*Myog* and *Myf6*), cell cycle genes, and transient myosin genes (*Myh3* and *Myh8*) and upregulation of *Cdkn2a*, suggesting a potential delay in cell cycle exit (Figure 4C). PAX7 expression corroborated the single-cell trajectory delay under hypothyroidism (Supplemental Figure 10). At 5 dpi, hypothyroid muscles displayed a higher density of PAX7^+^ cells compared with euthyroid, whereas PAX7^+^ counts were comparable at baseline and 14 dpi.

Furthermore, these cells exhibited increased expression of immune-related genes, including those involved in complement activation (*C1qa*, *C1qb*, *C1qc*), MHC class II antigens (*H2-Eb1*, *H2-Aa*, *H2-Ab1*), and cathepsin family members (*Ctsb*, *Ctss*). This profile is similar to the “immunomyoblast” population described by Oprescu et al. (13), indicating ongoing interactions with immune cells, which is absent in control cells after 5 days.

In uninjured hypothyroid myocytes (cluster 1), we observed upregulation of both transient and slow myosin genes, including the fast/oxidative isoform *Myh2* (note that *Myh2* is not displayed in the graph because of its high expression level) and the embryonic myosin *Myh3*, compared with control; and downregulation of genes related to skeletal muscle differentiation, including the fast glycolytic isoform *Myh4* (Figure 4C). At 14 dpi, hypothyroid myocytes (cluster 1) exhibited altered gene expression patterns related to terminal differentiation. Specifically, there was downregulation of *Myh4* (predominantly expressed in type IIb fast glycolytic fibers) and *Pvalb* (abundant in fast muscle fibers). Conversely, there was upregulation of *Myog*, *Myh1*, *Mymx*, and *Mymk* (involved in myocyte fusion), and *Car3* (highly expressed in mature slow fibers) (Figure 4C).

We then sought to determine the heterogeneity of myogenic populations as they transition through regeneration states. We performed trajectory analysis using Monocle 2 (https://cole-trapnell-lab.github.io/monocle-release/) on isolated control (Figure 4, D–H) and hypothyroid myogenic cells (Figure 4, I–L), which revealed 5 distinct states in control samples and only 3 in hypothyroid samples, supporting the reduced heterogeneity and complexity of the myogenic lineage in hypothyroid muscles. Both control and hypothyroid trajectories exhibited a similar hierarchical structure, branching into 3 main categories: *Myod1^+^* cells, cycling/proliferating cells, and differentiated myocytes (Figure 4, D and I, and Supplemental Datasets 10 and 11). However, 2 states were uniquely identified in control samples and were absent in hypothyroid samples (states 2 and 3, Figure 4, F, G, K, and L). These states, consisting of 2 small cell populations, were predominantly located within the myocyte branch at the 14-day time point (Figure 4, E–G). Comparative gene analysis indicated that these states represented cells transitioning from proliferation to differentiation, with a transcriptional profile suggesting involvement in stem cell niche regeneration (through expression of the quiescence markers *Pax7* and *Cd34* and the niche-related marker *Actc1*), and a progression from temporary to mature fibers (*Myh3*, *Myh8*, *Myh1*, *Myh4*) (Figure 4H and Supplemental Figure 11).

Collectively, these findings suggest that thyroid hormone is essential for maintaining the diversity and fidelity of myogenic differentiation during muscle regeneration, and that lack of thyroid hormone affects MuSC activation, proliferation, and differentiation.

### Hypothyroidism disrupts muscle stem cell cycle progression and alters muscle fiber composition during regeneration.

**Figure 5:**
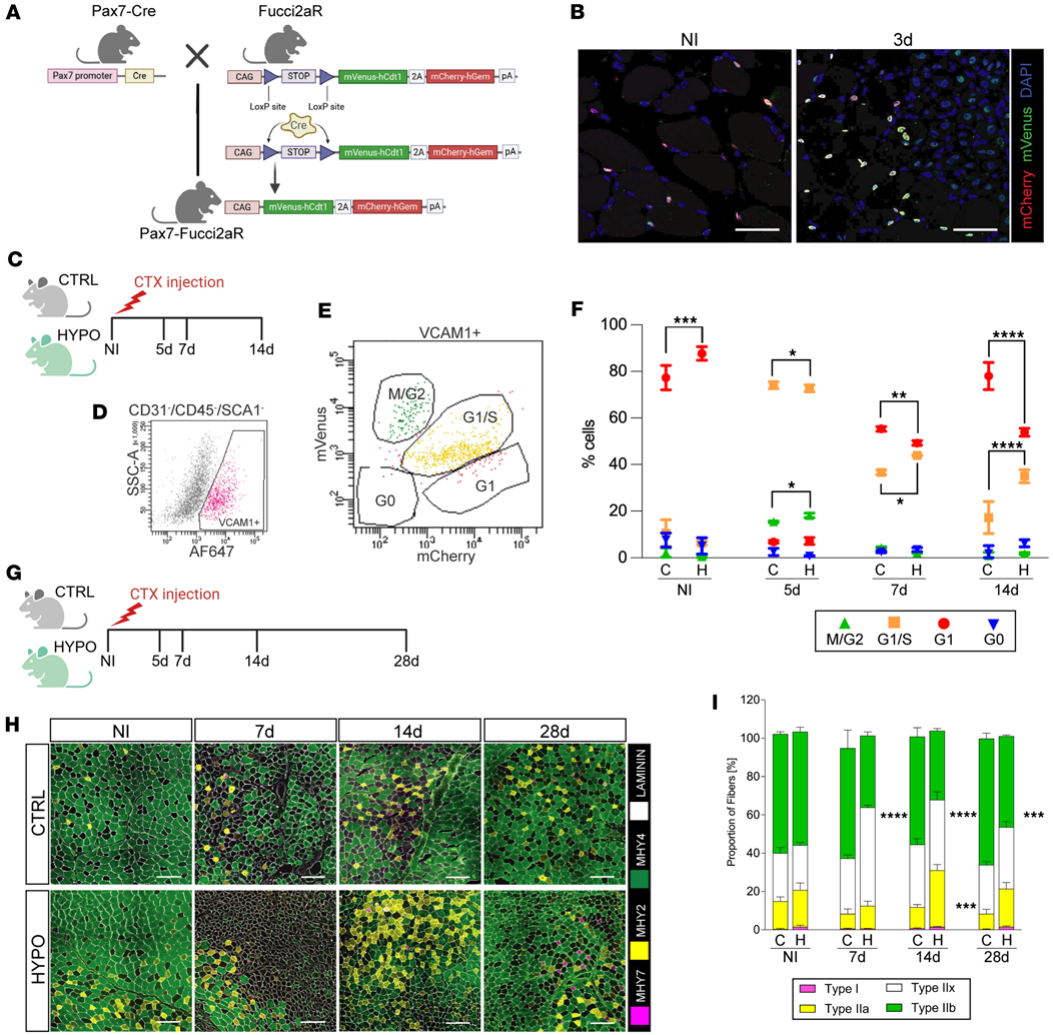
Hypothyroidism impairs muscle stem cell cycle progression and fiber type regeneration following injury. (**A**) *Pax7*-Fucci2aR mice are generated by crossing of Fucci2aR and mice expressing Cre recombinase under the *Pax7* promoter (Pax7-Cre). (**B**) Immunofluorescence images of uninjured and injured TAM 3 days after muscle injury, showing expression of the Fucci2aR reporter fluorophores mCherry (red) and mVenus (green). Nuclei in blue. Scale bars: 50 μm. (**C**) Experimental design overview. Primary cells were collected from TAMs isolated from control and hypothyroid mice before injury and 5, 7, and 14 days after cardiotoxin injury (*n* = 3 for each group) and subjected to flow cytometry analysis. Created in BioRender (Aguiari P, 2026, https://BioRender.com/dshnhgx). (**D**) Representative graphs showing selection of CD31^–^CD45^–^SCA1^–^VCAM1^+^ population by flow cytometry. (**E**) Representative quadrant of Fucci2aR reporter mVenus and mCherry expression in the VCAM1^+^ population: mVenus^+^ (M/G_2_, green), mCherry^+^ (G_1_, red), mVenus^+^mCherry^+^ (G_1_/S, yellow), and mVenus^–^mCherry^–^ (G_0_). (**F**) Superimposed dot plot graph showing proportion of MuSCs in different phases of the cell cycle in control and hypothyroid myogenic muscles before and 5, 7, and 14 dpi, as identified by flow cytometry analysis. *P < 0.05, **P < 0.01, ***P < 0.001, ****P < 0.0001. (**G**) Experimental design overview. TAMs were isolated from control and hypothyroid mice before injury and 5, 7, 14, and 28 days after cardiotoxin injury (*n* = 3 for each group) and subjected to histological analysis. Created in BioRender (Aguiari P, 2026, https://BioRender.com/ksp4qn5). (**H**) Immunofluorescence of TAM sections from control and hypothyroid mice at different time points before and after injury: myosin type I (MYH7, magenta), type IIa (MYH2, yellow), type IIb (MYH4, green), and laminin (white). Type IIx (MYH1) unstained. Scale bars: 100 μm. (**I**) Histogram plot showing proportion of different fiber types in TAM during regeneration at 0, 7, 14, and 28 days after cardiotoxin-induced injury. Data are presented as mean ± SEM. *n* = 3. Two-way ANOVA was used for statistical analysis. ***P < 0.001, ****P < 0.0001, in hypothyroid compared with time point–matched control fibers. The experiment was repeated 3 times independently under identical conditions. C, control; H, hypothyroid; CTRL, control; HYPO, hypothyroid.

To evaluate how hypothyroidism impairs regeneration by modulating MuSC proliferation and differentiation, we analyzed cell cycle phases in these cells using the FUCCI system. The system employs red (mCherry) and green (mVenus) fluorescent proteins fused to the cycling regulators CDT1 and geminin, respectively. These two proteins oscillate via posttranslational control: CDT1 accumulates in the G_1_ phase (mCherry^+^ nuclei, red), and when cells enter the S phase, it is degraded by ubiquitin-mediated proteolysis. At the same time, geminin accumulates from the early S phase through the M phase (mVenus^+^ nuclei, green). Both proteins are present during the G_1_/S transition (mCherry^+^mVenus^+^ nuclei, yellow), while in G_0_ and early G_1_, both reporters are absent, and cells have no fluorescence (19, 20). We generated mice with the FUCCI signal under the *Pax7* promoter (*Pax7*-Fucci mice) by breeding Fucci2AR-Gt(ROSA)26Sor^tm1(CAG-Venus/GMNN,-Cherry/CDT1)Jkn^ mice with *Pax7*-Cre (Pax7^tm1(cre)Mrc^/J) mice (Figure 5A). The FUCCI reporter system activates only in cells of the myogenic lineage (expressing *Pax7*) once they enter the cell cycle (Figure 5B). Three days after CTX-induced injury in the TAM of *Pax7*-Fucci mice, we observed a marked increase of mCherry^+^mVenus^+^ nuclei (yellow, G_1_/S phase) and mVenus^+^ nuclei (green, S/G_2_/M phase), indicating MuSC activation and proliferation (Figure 5B). During in vitro differentiation, *Pax7*-Fucci cells transitioned from green/yellow (G_1_/S/M phases) to red (G_1_ phase), confirming that red (G_1_) cells had exited the cell cycle and differentiated into myocytes (Supplemental Figure 12).

Cell cycle phase distribution in MuSCs was analyzed in hypothyroid and euthyroid mice at multiple time points after injury (0, 5, 7, and 14 days) (Figure 5C). For this, we used established markers to isolate quiescent/activated MuSCs (CD31^–^CD45^–^SCA1^–^VCAM^+^ cells) (21), and assessed the FUCCI signal in this population via flow cytometry (Figure 5, D and E).

Cell cycle analysis showed that, in uninjured hypothyroid muscle, MuSCs predominantly occupied the G_1_ phase (red), with a reduced presence in the S/G_2_/M phase (Figure 5F). After injury, hypothyroid MuSCs exhibited delayed cell cycle progression, with a lower proportion of cells in G_1_ and an increased proportion in S phase at days 7 and 14 (Figure 5F). These findings confirm that hypothyroidism affects MuSC proliferation and differentiation and may impair the regenerative progression.

To assess how hypothyroidism affects muscle fiber composition during regeneration and confirm that the differences seen in scRNA-seq in myocytes translate into phenotypical changes in mature fibers, we analyzed the whole-tissue fiber composition of regenerating TAM in both hypothyroid and euthyroid mice at 0, 7, 14, and 28 days and 2 months after injury (Figure 5, G and H). In euthyroid muscles, fiber composition remained consistent over time, with a prevalence of fast glycolytic type IIb fibers, and a temporary increase of type II fibers (Figure 5, H and I). Hypothyroid muscles, however, displayed a significant reduction in type IIb fibers at all time points after injury, accompanied by an increase in type IIx fibers at days 7, 14, and 28 after injury and in type IIa fibers at days 14 and 28. These alterations in fiber type and size indicate that hypothyroidism disrupts regeneration of normal muscle architecture and leads to lasting changes in muscle structure and potential function.

### Dysregulated immune and fibro-adipogenic progenitor responses in skeletal muscle regeneration during hypothyroidism.

Skeletal muscle regeneration is a highly coordinated process involving both myogenic and nonmyogenic cell populations, including immune cells and fibro-adipogenic progenitors (FAPs). Our scRNA-seq analysis revealed dynamic changes in these populations during regeneration in hypothyroid and control muscles (Figure 2, D and E). Given these observations, we investigated whether hypothyroidism contributes to alterations in the composition and behavior of these nonmyogenic populations.

**Figure 6:**
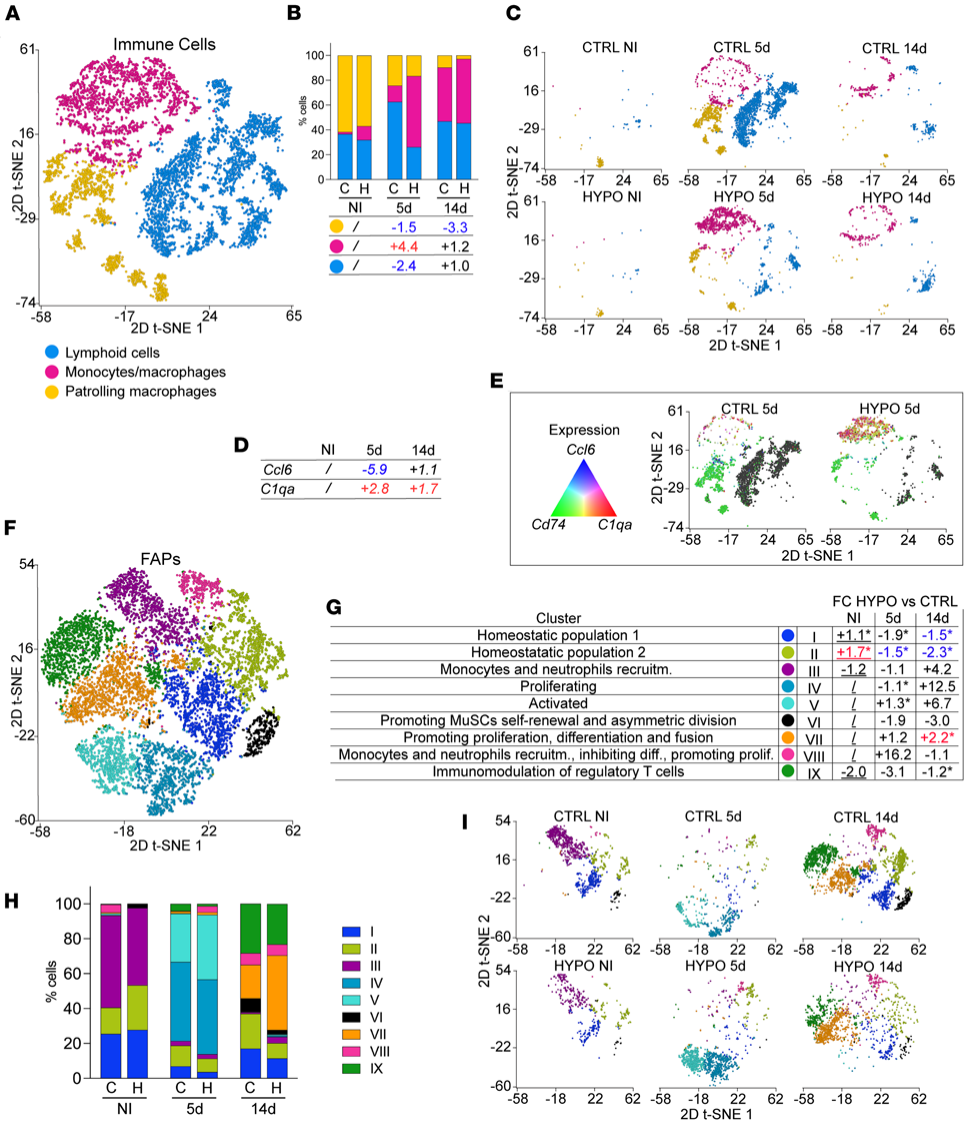
Thyroid hormone deficiency alters the immune and FAP landscape during skeletal muscle regeneration. (**A**) t-SNE visualization of integrated scRNA-seq datasets from control and hypothyroid immune cells, with clusters distinguished by color. (**B**) Proportions of cells (graph, top panel) in each cluster shown as a percentage of control and hypothyroid immune cell muscle before injury and 5 and 14 dpi. Bottom panel: Fold change in cell proportion in hypothyroid compared with control samples for each cluster. In uninjured (NI) datasets, the immune cell samples were too small to be compared (/). Number of cells for each sample: CTRL NI, 128; CTRL 5d, 3,381; CTRL 14d, 548; HYPO NI, 72; HYPO 5d, 2,052; HYPO 14d, 572. (**C**) t-SNE visualization of control and hypothyroid immune cell scRNA-seq data colored by cluster and grouped by time points. (**D**) Table showing fold change of the number of proinflammatory (*Ccl6^+^*) and antiinflammatory (*C1qa^+^*) macrophages in hypothyroid versus control muscles at 5 and 14 dpi. In uninjured (NI) datasets, the immune cell samples were too small to be compared (/). (**E**) t-SNE visualization of immune cells at 5 dpi in control and hypothyroid scRNA-seq datasets colored by expression of *Ccl6* (proinflammatory macrophages, blue), *C1qa* (antiinflammatory macrophages, red), and *Cd75* (patrolling macrophages, green). Cells negative for the 3 markers are shown in black. (**F**) t-SNE plot of integrated scRNA-seq datasets from control and hypothyroid FAPs, with clusters distinguished by color. (**G**) Table describing FAP clusters and their functional roles based on gene expression classification, cluster labeling, and fold change (FC) in cell number between hypothyroid and control. Roman numerals represent the cluster numbers used in the text; asterisks indicate FC values for clusters of more than 100 cells, which are underlined, FC > 1.5 in red; FC < –1.5 in blue. (**H**) Histogram bar graph showing relative abundance of FAPs and muscle before injury and 5 and 14 dpi. Number of cells for each sample: CTRL NI, 1,648; CTRL 5d, 919; CTRL 14d, 3,597; HYPO NI, 655; HYPO 5d, 1,784; HYPO 14d, 1,875. (**I**) Condition-specific t-SNE plots illustrating distribution of FAP clusters in control and hypothyroid muscles before injury and 5 and 14 dpi. C, control; H, hypothyroid; CTRL, control; HYPO, hypothyroid.

To examine immune cell dynamics, we selected 6,753 cells from immune clusters (myeloid, lymphoid, and mixed immune cell clusters; Supplemental Figure 13 and Supplemental Dataset 12) and performed unsupervised graph-based clustering. Differential gene expression analysis identified 17 distinct immune cell clusters that we broadly aggregated into 3 groups: monocytes and macrophages, patrolling macrophages, and lymphoid cells (Figure 6A and Supplemental Figure 13, A and B). Notably, the proportion of monocytes and macrophages was 4.4-fold higher in hypothyroid muscles at 5 dpi compared with controls (Figure 6, B and C). Given the critical role of pro- and antiinflammatory macrophages in muscle repair, we further analyzed the shifts in their proportions during regeneration. Proinflammatory (*Ccl6^+^*) cells were 5.9-fold less in the hypothyroid muscle 5 dpi, while the antiinflammatory (*C1qa^+^*) cells were 2.8-fold higher at the same time point (Figure 6, D and E).

To characterize FAP dynamics, we identified cell clusters exhibiting upregulation of FAP-associated genes across all datasets, yielding a total of 10,478 FAPs, which were subsequently subjected to subclustering analysis (Figure 6F, Supplemental Figure 14, and Supplemental Dataset 13). Graph-based clustering of the integrated FAP dataset identified 9 distinct subpopulations (clusters I–IX, Figure 6G). Based on differential gene expression analysis of known FAP markers (13, 22) (Supplemental Figure 14A), these clusters were classified according to their roles in myogenic regeneration (Figure 6G).

A different distribution of FAP subpopulations was observed between hypothyroid and control muscles across different time points (Figure 6, H and I). In uninjured muscle, we identified 2 homeostatic FAP populations (clusters I and II) characterized by genes associated with ECM production, turnover, and cytokine expression profiles known to inhibit MuSC differentiation and promote overall muscle growth. A third FAP population (cluster III) exhibited enrichment of genes encoding chemokines involved in monocyte and neutrophil recruitment. One of the 2 homeostatic FAP populations (cluster II) was increased in hypothyroid, noninjured (NI) muscles (Figure 6, H and I, and Supplemental Figure 14B). At 5 dpi, both control and hypothyroid muscle showed a reduction in homeostatic populations, with hypothyroid muscle exhibiting a greater decrease (Figure 6, H and I, and Supplemental Figure 14B). At this time point, a proliferative FAP population (cluster IV) and an activated FAP population (cluster V) were identified and comprised the larger percentage of the total FAPs. The activated FAP population is defined by expression of cytokine genes associated with recruitment of monocytes/macrophages and support of MuSC proliferation and differentiation. The proportion of activated FAPs increased 1.3-fold in hypothyroid muscles compared with controls 5 days after muscle injury (Figure 6, H and I, and Supplemental Figure 14B).

At 14 dpi, the homeostatic FAP populations responsible for ECM remodeling increased compared with 5 dpi. However, they were still underrepresented in hypothyroid muscles, showing 1.5-fold and 2.3-fold reductions compared with control muscles (Figure 6, H and I, and Supplemental Figure 14B). The percentage of proliferating and activated FABs was low at 14 days, with a trend of a higher proportion for the hypothyroid muscle compared with the control (Supplemental Figure 14B). Furthermore, a key FAP subpopulation (cluster VI) known to support MuSC self-renewal and asymmetric division (22) was 3-fold reduced (3.0-fold) in hypothyroid muscles, suggesting a long-term deficit in MuSC maintenance and regenerative potential (Figure 6, H and I, and Supplemental Figure 14B). In contrast, FAPs that promote MuSC differentiation and fusion (cluster VII) were overrepresented, exhibiting a 2.2-fold increase in hypothyroid muscles (Figure 6, G–I, and Supplemental Figure 14B).

Two FAP subpopulations, one aiding monocyte and neutrophil recruitment while inhibiting MuSC differentiation and promoting proliferation (cluster VIII) and another involved in regulatory T cell–mediated immunomodulation (cluster IX), were rare in hypothyroid and control muscles, but hypothyroid muscles showed higher cluster VIII and lower cluster IX 5 dpi (Figure 6, H and I, and Supplemental Figure 14B).

These findings suggest that hypothyroidism disrupts the normal progression of muscle repair, potentially leading to impaired regeneration due to dysregulated ECM remodeling, inflammatory responses, and MuSC maintenance.

### Direct and indirect actions of thyroid hormone receptor signaling during skeletal muscle regeneration.

**Figure 7:**
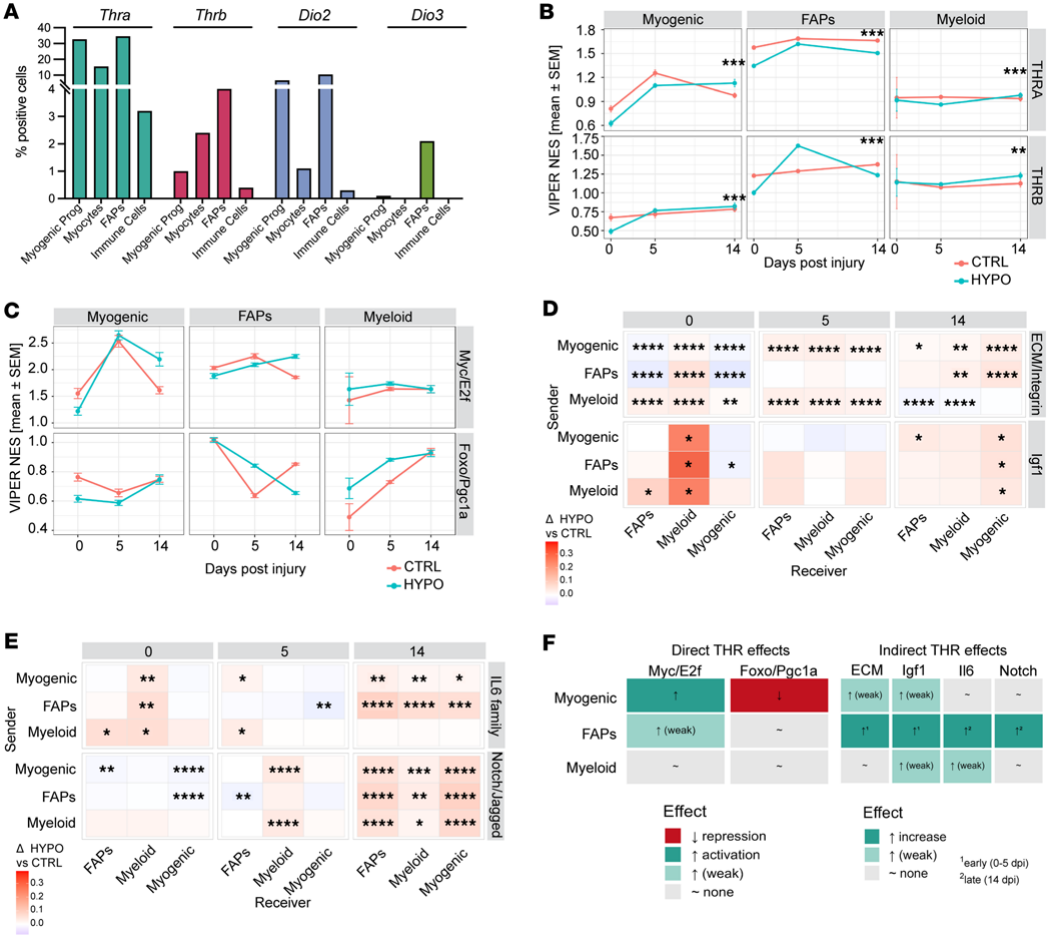
Thyroid hormone (T3) signaling and downstream regulatory programs during muscle regeneration. (**A**) Percentage of cells in each population expressing *Thra*, *Thrb*, *Dio2*, and *Dio3*. (**B**) Temporal modeling of *Thra/b* activity (generalized additive model [GAM], *k* = 3). GAM-like fits of VIPER-inferred normalized enrichment scores (NESs; mean ± SEM) for *Thra* (top) and *Thrb* (bottom). Lines show temporal trends; ribbons denote ± SEM. Asterisks indicate statistically significant divergence between CTRL and HYPO trajectories (**P < 0.01, ***P < 0.001, ANOVA comparing GAM smooths). (**C**) Line plots show CollecTRI-VIPER NESs for proliferative (*Myc*/*E2f*) and oxidative (*Foxo*/*Ppargc1a*) transcriptional programs across cells. Each line represents the average program activity per condition and time point. In FAPs, hypothyroidism suppressed the *Myc*/*E2f* cycling module at baseline and 5 days post-injury (dpi) but reactivated it at 14 dpi, while the oxidative *Foxo*/*Ppargc1a* program showed the opposite trend: early induction and late repression. Myogenic cells showed a similar inversion of proliferative and oxidative trajectories, whereas myeloid populations exhibited only a transient oxidative increase at 5 dpi. (**D** and **E**) Tile heatmap displaying hypothyroid-induced changes in LR interactions stratified by sender (rows) and receiver (columns) for each pathway family across the 3 regeneration stages. The color scale encodes the effect size (HYPO – CTRL). This figure resolves the directionality of communication. Asterisks indicate FDR-adjusted Wilcoxon’s *P* values (*P < 0.05, **P < 0.01, ***P < 0.001, ****P < 0.0001). (**F**) CollecTRI-VIPER transcriptional modeling and LR pathway analysis were integrated to compare direct (*Thr* regulon) and indirect (ECM, IGF-1, IL-6, Notch) effects across myogenic, FAP, and myeloid lineages. β-Coefficients were summarized as qualitative activation (arrow up), repression (arrow down), or weak effects at early and late stages of regeneration.

To dissect how thyroid hormone regulates both myogenic and nonmyogenic compartments during repair, we quantified *Thra*, *Thrb*, *Dio2*, and *Dio3* expression across major populations (Figure 7A). *Thra* was prevalent in FAPs and myogenic progenitors, *Thrb* was scarce, and *Dio2* was enriched in stromal and myogenic cells, indicating multiple potential entry points for T3 availability and receptor-mediated activation within skeletal muscle.

Next, we used the VIPER (Virtual Inference of Protein-activity by Enriched Regulon analysis) algorithm, which allows computational inference of protein activity from gene expression profile data, to infer *Thra* and *Thrb* regulon activity. We applied the CollecTRI-VIPER framework on our scRNA-seq data (validated by 78% concordance with DoRothEA; Supplemental Figure 15). Unlike differential expression analyses that measure changes in individual gene abundance, VIPER infers functional transcription factor activity from the coordinated regulation of its downstream targets (the regulon) (23, 24). Thus, “THR activity” here refers to the inferred activation state of the *Thra/b* regulons, independent of *Thra* or *Thrb* mRNA expression.

Temporal modeling of normalized enrichment scores across lineages revealed distinct, cell-specific trajectories (Figure 7B). In the control muscle, *Thra/b* activity rose sharply between 0 and 5 dpi and normalized by 14 dpi, particularly in myogenic cells and FAPs. Under hypothyroidism, this induction was blunted or delayed: *Thra* activity remained low, while *Thrb* displayed a transient rise 5 dpi followed by decline. Myeloid cells showed minimal change, consistent with indirect modulation via paracrine cues. These divergent trajectories were also confirmed by bootstrapped confidence intervals (Supplemental Figure 16, A and B).

To evaluate how thyroid hormone signaling integrates with regenerative control, we focused on *Myc*/*E2f* and *Foxo*/*Ppargc1a* regulons (Figure 7C and Supplemental Figure 17A), which respectively govern proliferative expansion and oxidative maturation of myogenic precursors. As for *Thra/b*, these activities represent inferred transcriptional output of the entire program, not expression of individual pathway genes. VIPER infers the coordinated activation state of transcriptional modules from the collective behavior of their targets, allowing assessment of how proliferative and oxidative programs are transcriptionally engaged in each condition. Under euthyroid conditions, proliferative activity peaked early, and oxidative programs rose later, marking the normal switch from expansion to differentiation (25, 26). Hypothyroidism inverted this pattern: proliferative *Myc*/*E2f* activity was suppressed at baseline and early phases but markedly increased by 14 dpi, whereas oxidative activity peaked prematurely at 5 dpi and then declined. The apparent discrepancy with the high number of cycling cells observed at 5 dpi under hypothyroidism reflects a decoupling between cell abundance and transcriptional drive: although more cells enter the cycling compartment under hypothyroidism, their *Myc*/*E2f* regulon activity is reduced, indicating inefficient or incomplete proliferative engagement. *Thra/b* activity correlated positively with *Myc*/*E2f* and negatively with *Foxo*/*Ppargc1a* scores (Supplemental Figure 17B), demonstrating that thyroid hormone directly synchronizes proliferative and metabolic transitions in regenerative lineages.

Finally, ligand-receptor (LR) mapping identified remodeling of intra- and inter-population paracrine networks under hypothyroidism (Figure 7, D and E, and Supplemental Figures 18 and 19). Hypothyroidism preferentially altered ECM/integrin, IGF-1, IL-6 family, and Notch/Jagged signaling, with distinct temporal patterns (Supplemental Figure 18). At baseline, matrix-integrin signaling between FAPs and myocytes was reduced. By day 5, however, it became enriched in myogenic-myeloid exchanges, while FAPs acted largely as receivers. By day 14, FAPs emerged as predominant matrix-signal senders, particularly toward myogenic cells, establishing reciprocal FAP-myogenic communication (Figure 7D and Supplemental Figure 19). IGF-1 and IL-6 pathways showed transient amplification, and Notch/Jagged interactions became globally activated at 14 dpi (Figure 7, D and E).

To dissect direct nuclear versus indirect paracrine influences of thyroid hormone signaling, we performed linear modeling of CollecTRI-VIPER transcriptional programs (*Myc*/*E2f*, *Foxo*/*Ppargc1a*) as a function of THR regulon activity and LR pathway scores (ECM, IGF-1, IL-6, Notch). T3-THR signaling orchestrates regeneration through both direct transcriptional and indirect paracrine mechanisms (Figure 7F). In myogenic cells, *Thr* activity was associated positively with *Myc*/*E2f* proliferative programs and inversely with *Foxo*/*Ppargc1a* oxidative programs. In FAPs, *Thr* links were weaker and largely mediated through paracrine remodeling, characterized by early ECM/IGF-1 and late IL-6/Notch pathway activation. Myeloid populations displayed only mild or context-dependent effects, consistent with secondary modulation (Figure 7F). Together, these data establish that thyroid hormone drives regeneration by synchronizing myogenic transcriptional programs with temporally coordinated stromal-immune signaling dynamics. In hypothyroidism, this coordination is disrupted, delaying *Thr*-dependent activation and prolonging compensatory stromal and cytokine signaling, ultimately impairing regenerative timing and efficiency.

## Discussion

Skeletal muscle regeneration is a complex, highly regulated process that relies on the coordinated interplay between MuSCs, FAPs, and immune cells to restore tissue integrity following injury (13, 27).

Our study reveals that hypothyroidism profoundly impairs skeletal muscle regeneration following cardiotoxin-induced injury in mice by reducing myogenic-lineage diversity, affecting MuSC cell cycle, and altering nonmyogenic cell contributions.

Using scRNA-seq and the *Pax7*-Fucci model, we demonstrate that hypothyroid muscles exhibit smaller myofibers persisting up to 2 months after injury, a shift toward slower oxidative fiber types, and defective cellular coordination, offering insights into thyroid hormone’s role in repair processes.

The observed reduction in myofiber size and delayed recovery align with prior mouse models of disrupted thyroid hormone signaling, such as THRA-PV mutants (9, 28) and D2/D3 knockouts (10, 29, 30), which show impaired regeneration and sarcopenia-like phenotypes.

This study examines how thyroid hormone deficiency impacts skeletal muscle regeneration. We observed differences in muscle-resident cell populations between hypothyroid and control muscles, particularly among myogenic progenitors, FAPs, and immune cells, which could explain impaired regeneration.

In the euthyroid myogenic cell population we identified a distinct quiescent/activated MuSC cluster that was markedly diminished in hypothyroid samples, which instead showed enrichment for immediate-early genes such as *Fos* and *Jun*, markers of a “pre-activated” but nonproductive state (31, 32). It is possible that hypothyroidism might shift myogenic cells into a “pre-activated” state rather than promoting maintenance of the quiescent state. This transcriptional signature suggests that MuSCs in hypothyroid muscles are poised for activation but fail to efficiently progress through the myogenic program during regeneration. These findings align with the knowledge that T3 directly controls *Myod1* expression, which is necessary to induce MuSC activation and entry into the cell cycle (5, 6). Then, T3 promotes cell cycle exit and expression of an array of muscle-specific genes during myoblast differentiation, from *Myog* in the immature myotube to myosin heavy chain isoforms in the mature myofiber (5), as observed in mice lacking D2 (the enzyme converting T4 to T3) that exhibit defective differentiation (10).

Cell cycle dysregulation further underscores hypothyroidism’s impact. The surge in proliferative MuSCs at 14 dpi, coupled with the prolonged G_1_/S arrest, indicates a bottleneck in transitioning to differentiation. Upregulation of *Cdkn2c*, a senescence marker linked to aging muscle (33), in hypothyroid proliferative clusters suggests that thyroid hormone deficiency may induce a senescence-like state, stalling myogenesis. The selective increase of PAX7^+^ cells at 5 dpi in hypothyroid muscle most parsimoniously reflects delayed progression from PAX7^+^ progenitors toward MYOD/MYOG states, rather than a baseline expansion of the stem pool. This interpretation is supported by the concordant depression of *Thra* activity, FUCCI-inferred G_1_/S delay, and the early-phase signaling milieu (reduced Notch tone, altered ECM/integrin directionality) that is less permissive for timely commitment. Together, these data show that thyroid hormone is required for both timely cell cycle progression and exit, which are essential for successful MuSC differentiation and muscle repair. Moreover, the reduction in fast glycolytic type IIb fibers (MYH^+^) and increase in type IIa/IIx fibers (MYH1^+^) in hypothyroid regenerated muscles corroborate thyroid hormone’s established influence on fiber type specification, which favors fast-twitch profiles in euthyroid states (5, 34). Our histological findings confirm the transcriptomics data of altered muscle fiber composition in hypothyroid muscle after injury. Although our data highlight MuSC dysfunction as a central mechanism underlying defective regeneration in hypothyroidism, we cannot exclude direct effects of thyroid hormone deficiency on myonuclei. Thyroid hormones regulate metabolic and fiber type programs within mature myofibers, and the persistent shift toward oxidative fiber types we observed may reflect a combination of impaired MuSC differentiation and altered myonuclear gene expression. Future studies will be essential to disentangle MuSC-intrinsic versus myofiber-autonomous contributions to hypothyroid myopathy.

Beyond MuSC-intrinsic defects, hypothyroidism also perturbs the activity of nonmyogenic cell types essential for regenerative success: immune cells and FAPs (1). In healthy regeneration, a timely transition from proinflammatory to antiinflammatory macrophages is essential for clearing necrotic tissue, activating MuSCs, and guiding subsequent differentiation and remodeling (1, 35, 36). Our analysis of immune cell clusters demonstrated a substantial elevation in monocytes and macrophages in hypothyroid muscles at 5 dpi, a time point corresponding to peak inflammatory response. However, this increase was not accompanied by a proportional rise in proinflammatory (*Ccl6^+^*) macrophages. Instead, hypothyroid muscle showed a marked reduction in proinflammatory cells and a concomitant elevation in antiinflammatory macrophages.

Parallel to these immune alterations, we observed striking differences in FAP composition and temporal progression. FAPs play essential roles in ECM remodeling and the modulation of MuSC fate through paracrine signaling (37). In uninjured muscle, we identified two homeostatic FAP populations, which maintain ECM integrity and secrete cytokines. However, in uninjured hypothyroid muscle, there was an increased presence of one homeostatic FAP subpopulation, suggesting a compensatory adaptation or chronic ECM remodeling even in the absence of injury.

Following injury, the decline of homeostatic FAPs and rise of proliferative and activated FAPs are expected (38). However, the increase in activated FAPs at day 5 in hypothyroid muscle may reflect an exaggerated inflammatory or stress response. These activated FAPs, while supporting MuSC activation and differentiation, may also contribute to premature or dysregulated tissue remodeling if not properly resolved. By day 14, the expected reemergence of homeostatic FAPs was blunted in hypothyroid muscle, indicating a persistent impairment in ECM regulation and support for MuSC renewal. Moreover, hypothyroid muscle exhibited a substantial reduction of a FAP subpopulation that promotes MuSC self-renewal and asymmetric division. In contrast, FAPs promoting MuSC differentiation and fusion were overrepresented, which may suggest a delay in skeletal muscle differentiation.

The presence of distinct FAP populations involved in immune modulation and myeloid cell recruitment further highlights the intricate crosstalk between stromal and immune compartments. Altered proportions of these populations in hypothyroid muscle may exacerbate the dysregulated immune landscape, contributing to an overall suboptimal regenerative environment.

Integrated transcriptional and LR analyses revealed that T3 governs muscle regeneration through dual mechanisms. Directly, *Thra/b* activities promote *Myc*/*E2f*-driven proliferation (39) while restraining *Foxo*/*Ppargc1a* oxidative programs (40) in myogenic cells, ensuring proper temporal coordination of growth and metabolism. Indirectly, T3 shapes paracrine communication: early regeneration is dominated by myeloid-to-FAP IL-6/IGF-1 signals, whereas later FAPs become major senders of ECM, IL-6, and Notch cues toward myogenic and immune compartments. Under hypothyroidism, this sequence is delayed. Thus, thyroid hormone synchronizes intrinsic (transcriptional) and extrinsic (paracrine) programs essential for efficient tissue repair, and its loss destabilizes both axes, leading to maladaptive regeneration. Our findings parallel observations in other systems, such as glial-to-neuron paracrine T3 signaling in the brain (41) and T3-dependent remodeling of epithelial stromal compartments during amphibian metamorphosis, where thyroid hormone action in connective tissue is necessary to instruct stem cell behavior in adjacent epithelium (42). In conclusion, our data establish thyroid hormone as a fundamental regulator of skeletal muscle regeneration, acting through dual modes: directly on myogenic transcriptional programs, and indirectly by reshaping paracrine communication between FAPs, immune cells, and MuSCs. These insights provide a mechanistic framework for the muscle pathology observed in hypothyroid conditions and highlight thyroid hormone as a key targetable axis for improving muscle repair in endocrine or age-associated myopathies.

## Methods

### Sex as a biological variable.

All experiments were conducted using male mice. This decision was based on the evidence that the majority of studies investigating skeletal muscle regeneration and thyroid hormone signaling use male animals, allowing for direct comparison with existing literature and minimizing biological variability associated with sex hormone fluctuations. Future studies will be necessary to determine whether the observed effects of hypothyroidism on myogenic-lineage diversity and regeneration dynamics are conserved in females.

### Mice.

The *Pax7*-Fucci mouse model was generated by breeding of a Fucci2aR mouse [B6;129-Gt(ROSA)26Sor^<tm1(Fucci2aR)Jkn^; stock RBRC06511], purchased from Riken, with a *Pax7*-Cre (Pax7^<tm1(cre)Mrc>^/J, strain 010530, The Jackson Laboratory) expressing Cre recombinase under the *Pax7* promoter. *Pax7*-Fucci mice heterozygous for Cre were used for immunofluorescence and flow cytometry experiments, while *Pax7*-Fucci mice negative for Cre were used for transcriptomic experiments. *Pax7*-Fucci mice between the ages of 3 and 5 months were used for the experiments (total 90 mice).

### Hypothyroidism and muscle injury.

Hypothyroidism was induced by feeding of a commercially available iodine-deficient diet containing propylthiouracil (PTU; 0.15% wt/wt) (TD.08259, Envigo Teklad) to 3- to 5-month-old *Pax7*-Fucci mice (43). Control mice received a matched diet (TD.120461, Envigo Teklad) containing 0.7 mg/kg iodine (from potassium iodate) and no PTU. This ensures that nutritional differences between groups are limited to iodine and PTU. The diets began on day 1, and mice were housed in freshly cleaned cages to prevent iodine contamination from prior environments. The experimental diet was maintained for 3 weeks before muscle injury and continued throughout the study (up to 2 months after injury) to sustain the hypothyroid condition.

After 3 weeks, the right TAM of each mouse was injected with cardiotoxin (CTX; 50 μg/mL; Sigma-Aldrich) to cause injury. Mice were euthanized at different time points (0, 4, 5, 7, 14, and 28 days and 2 months) after the injury for further analysis.

### Serum triiodothyronine assay.

Serum triiodothyronine (T3) concentrations were quantified using a commercial enzyme immunoassay kit (catalog T3043T-100, Calbiotech) according to the manufacturer’s instructions. For each mouse, T3 was measured at 3 time points: prior to initiation of the low-iodine/PTU diet (baseline), after 3 weeks on the diet immediately before CTX injection, and 2 weeks after injury. Serum samples and T3 standards were assayed in duplicate. Optical density was read at 450 nm using a microplate spectrophotometer, and T3 concentrations (ng/mL) were calculated from a standard curve generated with kit-provided calibrators.

### Immunofluorescence and histology.

To study TAMs using immunofluorescence, the isolated muscles were first frozen in isopentane cooled by liquid nitrogen. Thin sections, 5 μm thick, were then cut using a Leica Cryostat CM1950 (Leica Biosystems). The sections were dried at room temperature for 10 minutes and treated with a solution of 2% bovine serum albumin (BSA) in phosphate-buffered saline (PBS) for 1 hour to prevent unwanted binding. Next, the sections were incubated overnight at 4°C with the antibody (Table 1). After washing, the sections were incubated with a secondary antibody (Table 1) for 30 minutes at room temperature. Finally, the sections were mounted using VECTASHIELD Antifade Mounting Medium with DAPI (H1200, Vector Laboratories), and images were taken with a Leica DM5500 B microscope fitted with a DFC360 FX camera (Leica Biosystems). For PAX7 staining, whole-tissue pictures were collected with a Leica DMI6000 B fluorescence microscope equipped with a Leica DFC300 FX camera. The total number of PAS7-positive DAPI-positive nuclei was detected in QuPath software (47) using the Detect Cells function.

For histological analysis of *Pax7*-Fucci mice, isolated TAMs were fixed in 4% paraformaldehyde for 2 hours at room temperature. The fixed muscles were then embedded in paraffin wax, and 5 μm sections were cut using a Leica microtome RM2235 (Leica Biosystems). These sections were deparaffinized and mounted with DPX Mountant for histology (44581, Fluka, Sigma-Aldrich) for microscope observation. Images were captured using the same Leica DM5500 B microscope equipped with a DFC360 FX camera (Leica Biosystems).

### Fiber typing and minimum Feret diameter.

Fiber typing and minimum Feret diameter were determined by quadruple immunofluorescence using monoclonal antibodies against myosin heavy chain type I (MYH7), type IIa (MYH2), type IIb (MYH4), and laminin (44) (Table 1). Briefly, cryosections were dried for 10 minutes at room temperature and incubated with the Mouse Ig Blocking Reagent (Mouse on Mouse detection kit, Vector Laboratories) for 1 hour at room temperature, and then with primary antibodies for 45 minutes at 37°C. Sections were incubated with secondary antibodies (Table 1) for 45 minutes at 37°C and then mounted with ProLong Glass Antifade Mountant (P36948, Thermo Fisher Scientific). Whole-tissue pictures were collected with a Leica DMI6000 B fluorescence microscope equipped with a Leica DFC300 FX camera. Single-color images were merged to obtain a whole-muscle reconstruction. The laminin channel was inverted with Fiji ImageJ software (45), and individual fibers were segmented with CellPose (46) in QuPath software (47). For the total number of fibers, the number of fibers positive for each myosin marker, and the minimal Feret diameter, all the fibers from the tissue sections were measured.

### scRNA-seq and analysis.

A whole-muscle single-cell suspension was obtained using a modified version of a previously described protocol (21). Briefly, TAMs were isolated from 3- to 5-month-old male control and hypothyroid mice before injury (day 0) and 5 and 14 days after CTX-induced injury (*n* = 3 per group). Animals were euthanized by CO_2_ exposure, and hind-limb muscles were removed. TAMs were cleaned, minced, and then digested in RPMI medium 1640 (catalog 11875-093, Gibco) containing 0.2% collagenase type II (catalog LS004176, Worthington) and 0.55 U/mL Dispase (catalog 17105-041, Gibco) for 30 minutes at 37°C. After digestion, tissue was passed through a 16-gauge needle and filtered through a 100 μm and a 40 μm mesh to obtain a single-cell suspension. Cells from each mouse were pooled in one sample (*n* = 3). Removal of red blood cells and dead cells was performed using, respectively, the Red Blood Cell Lysis Solution (130-094-183) and the Dead Cell Removal Kit (130-090-101; both from Miltenyi Biotec) following the manufacturer’s recommendations.

Approximately 5,000 cells were captured on a 10x Chromium device using a 10x V3 Single Cell 3′ Solution kit (Chromium Single Cell 3′ Reagent kit V3 Chemistry, catalog PN-1000092, 10x Genomics). All protocols were performed following the manufacturer’s instructions. Final sequencing libraries were analyzed on a High Sensitivity DNA Chip (Agilent, catalog 5067-4626) to determine the library size; final library concentrations were determined using a Qubit High Sensitivity DNA Assay Kit (catalog Q32854, Thermo Fisher Scientific). Libraries were sequenced with the paired end setting of 101–101 with 8 cycles of index read on an Illumina NovaSeq 6000 platform. Approximately 30,000 reads per cell were sequenced.

The scRNA-seq data were analyzed with Partek Flow software v11.0 (Illumina, Inc.). Fastq sequencing files were aligned to the *Mus musculus* reference genome mm39 using Cell Ranger Gene Expression aligner (10x Genomics), and the transcript abundance was estimated using the Partek Flow algorithm based on GENCODE (https://www.gencodegenes.org/) release M30 transcript model with default settings. For the single-cell QA/QC, we applied threshold to filter out low-quality cells based on the total unique molecular identifier (UMI) count (<100 or >8,000), detected gene count (<500 or >50,000), and mitochondrial UMI proportion (1%–15%). All samples were processed jointly and subjected to identical mitochondrial quality control filtering in Partek Flow, using a uniform threshold of 1%–15% mitochondrial reads. The 15% was selected to retain metabolically active myogenic cells, which physiologically exhibit higher mitochondrial transcription. Read counts per gene in all samples were normalized by log_2_ counts per million and addition of 1 for values of zero. We applied top 20 principal components to generate t-distributed stochastic neighbor embedding (t-SNE) with default settings. Unsupervised graph-based clustering was performed on the integrated datasets with different resolutions (0.3 for the whole tissue, 1 for the myogenic lineage, 1 for immune cells, and 0.5 for FAPs) after multiple trials to better describe the different populations identified.

### CollecTRI-VIPER analysis of THRα/β activity.

scRNA-seq data were analyzed to infer THR activity. Mouse regulons for *Thra* and *Thrb* were obtained from CollecTRI (23), converted to VIPER regulon objects, and scored with the VIPER algorithm on the normalized expression matrix (24), as implemented in the decoupleR Bioconductor package (48). For each condition × time point normalized enrichment scores (NESs) were computed per cell for *Thra* and *Thrb* regulons. Group-level differences (hypothyroid vs. control) were assessed across cells by Wilcoxon’s rank-sum tests, and false discovery rates were reported for descriptive purposes. Because each dataset corresponds to pooled nuclei from 3 mice processed as one library, these statistics quantify within-sample enrichment among cells and do not reflect biological replication. To check reproducibility we repeated the analysis using DoRothEA regulons (levels A–E) (49) converted to VIPER objects. Program-level activities for *Myc*/*E2f* and *Foxo*/*Ppargc1a* regulons were calculated using the same matrix, and cell-wise Spearman’s correlations were used to assess covariation between *Thra/b* NESs and program scores.

### Temporal modeling and bootstrapping.

Temporal trajectories were modeled using generalized additive models with cubic regression splines (*k* = 3) (50, 51). For each receptor × cell type, an ANOVA compared models with shared versus condition-specific smooths to test whether control (CTRL) and hypothyroid (HYPO) trajectories differed significantly. Model-based predictions and 95% confidence intervals were extracted for visualization, and results were summarized as *F* statistics and nominal *P* values (Benjamini-Hochberg adjusted where indicated). Plots show fitted trajectories with ribbons (95% confidence interval) overlaid with observed means ± SEM. Bootstrapping (1,000 resamples) of per-cell NES values estimated intra-sample stability and 95% confidence intervals (52).

### Ligand-receptor interaction analysis.

Intercellular communication was profiled with OmnipathR (53, 54). The mouse ligand-receptor network (intercell_network, organism = 10,090) was filtered for expressed genes and used to compute edge interaction scores (mean ligand/receptor expression) for each sender-receiver pair at 0, 5, and 14 dpi. Differential LR activity (Δ = HYPO – CTRL) was aggregated into major pathway families (ECM/integrin, IL-6, IGF-1, Notch/Jagged) and visualized as global heatmaps. For each lineage (myogenic, FAPs, myeloid) and CollecTRI-VIPER program (*Myc*/*E2f*, *Foxo*/*Ppargc1a*), we performed standardized linear modeling at the single-cell level to estimate direct transcriptional versus indirect paracrine influences of thyroid hormone receptor activity. Direct (nuclear) effects were assessed by modeling of CollecTRI-VIPER transcriptional programs (*Myc*/*E2f*, *Foxo*/*Ppargc1a*) as a function of THR regulon activity across cell lineages. Indirect (paracrine) effects were evaluated by correlation of ECM, IGF-1, IL-6, and Notch LR pathway scores with the same transcriptional programs across time (0–14 dpi). β-Coefficients were summarized qualitatively as activation (arrow up), repression (arrow down), weak (weak), or none, and visualized as categorical heatmaps indicating early (0–5 dpi) and late (14 dpi) interactions.

### Primary myoblast isolation, cell culture, and differentiation.

Primary murine myoblasts were isolated from 3-month-old male euthyroid *Pax7*-Fucci mice using the protocol described above. For in vitro myogenic differentiation, cells were resuspended in DMEM/F12 with 10% horse serum (HS), 20% FBS, and 0.2% Primocin (catalog ant-pm-1, InvivoGen) and placed into a noncoated 100 mm tissue-culture Petri dish for 3 hours to let fibroblasts attach. Cells in suspension were then transferred to tissue culture Ibidi chamber slides coated with laminin-511 (Laminin iMatrix-511 E8, Amsbio) and incubated at 37°C and 5% CO_2_. After 48 hours, medium was replaced with fresh medium. Further medium changes were performed by replacement of 50% of the medium every 48 hours to ensure the maintenance of “conditioning” factors for all the duration of the experiments. To induce myogenic differentiation, DMEM/F12 medium with 2% HS was added when the cells were approximately 70% confluent. FUCCI signal was acquired with a Leica DMI6000 microscope equipped with a DFC295 B camera.

### Flow cytometry.

We analyzed murine myoblasts freshly isolated from 3-month-old control and hypothyroid *Pax7*-Fucci mice before and after CTX-induced injury (0, 5, 7, and 14 days). For the analysis of the CD31^–^CD45^–^SCA1^–^VCAM1^+^ populations, fresh cells were blocked in 2% BSA solution in PBS for 10 minutes and incubated with CD31, CD45, and SCA1 antibodies (Table 1) for 30 minutes at 4°C. Cells were then incubated with Anti-Rat IgG MicroBeads (130-048-502, Miltenyi Biotec) for 15 minutes at room temperature following the manufacturer’s instructions, and depletion of CD31^+^CD45^+^SCA1^+^ cells was carried out with LS columns (catalog 130-042-401, Miltenyi Biotec). The negative fraction was then incubated with APC-conjugated CD106/VCAM1 antibody (Table 1) for 30 minutes at 4°C and analyzed on a BD FACSymphony using the FACSDiva software (BD Biosciences).

The gating strategy was as follows: cells were first gated based on forward and side scatter (FSC/SSC) to exclude dead cells, then gated for FSC-width (FSC-W) by FSC-height (FSC-H) and SSC-width (SSC-W) by SSC-height (SSC-H) to exclude potential doublets. Final sorting gates were established based on the unstained control for each sample. Quantitative comparisons of cell populations were based on percentages of gated events.

### Statistics.

For imaging and flow cytometry experiments, data were imported and analyzed using GraphPad Prism version 10.0.0 (GraphPad Software). Comparisons were made by 1-way or 2-way ANOVA followed by Šidák’s multiple-comparison test. Data were plotted using GraphPad Prism and annotated with the results of the corresponding test. scRNA-seq data were processed using Partek Flow software v11.0. Differential gene expression between groups was analyzed using gene Poisson regression, with multiple testing corrected via the false discovery rate (FDR) method. Genes were considered significantly differentially expressed if they had an adjusted *P* value less than 0.01 and an absolute fold change greater than 2. Gene Ontology analyses were performed using GENEONTOLOGY (https://geneontology.org; database doi: 10.5281/zenodo.12173881; reference list: *Mus musculus*) through the PANTHER overrepresentation test to identify enriched biological processes.

For the single-cell analyses of *Thr* activity and cell-cell communication, VIPER NESs were compared between CTRL and HYPO samples within each cell type and time point using Wilcoxon’s rank-sum tests, and *P* values were Benjamini-Hochberg (FDR) adjusted. Competing TF program scores and LR pathway scores were tested in the same manner. Associations between per-cell VIPER scores (*Thra* or *Thrb*) and pathway-specific LR module scores (e.g., ECM/integrin, Notch) were assessed by linear regression. All scRNA-seq statistical analyses were performed in R (v4.5.1) using Seurat, decoupleR/OmnipathR, and ggplot2.

Data are presented as mean ± SEM. *P* values less than 0.05 were considered statistically significant. Sample sizes were based on preliminary data and standard practice in the field. All experiments were repeated independently at least twice with consistent results. Exact *n* values (biological replicates) for each experiment are indicated in the figure legends.

### Study approval.

All animal studies were performed in accordance with experimental protocols approved by the Institutional Animal Care and Use Committee of Children’s Hospital Los Angeles. Animal handling was performed according to the institutional guidelines for laboratory animal care at Children’s Hospital Los Angeles. The authors complied with the Animal Research: Reporting of In Vivo Experiments (ARRIVE) guidelines.

### Data availability.

The single-cell RNA sequencing data generated in this study were deposited in the Gene Expression Omnibus (GEO) database under accession number GSE311479 and are publicly accessible in compliance with MINSEQE guidelines. Supporting data values for all figures in the main article and supplement are provided in a separate file titled Supporting Data Values, with individual tabs corresponding to each figure panel. Additional data supporting the findings of this study are available upon reasonable request.

## Author contributions

AM and LP conceived and supervised the project. PA designed and conducted experiments and acquired and analyzed data. VV assisted with the scRNA-seq experiments and bioinformatics analysis. YYL assisted with data visualization and marker gene identification. GC assisted with statistical analysis and data interpretation. GAB and YYL contributed to data interpretation and project development. PA and AM wrote the manuscript with input from all authors. All authors reviewed and approved the final version of the manuscript.

## Funding support

- VA Merit Grant BX006245 (to AM).
- The GOFARR Foundation.
- The Schenkman family (to LP).
- Two Pilot Core Grants from the Children’s Hospital Los Angeles Spatial Biology and Genomics Core (to LP).
- VA Merit Grant BX001966 (to GAB).

## Supplementary Material

Supplemental data

Supplemental data sets 1-13

Supporting data values

## Figures and Tables

**Table 1:**
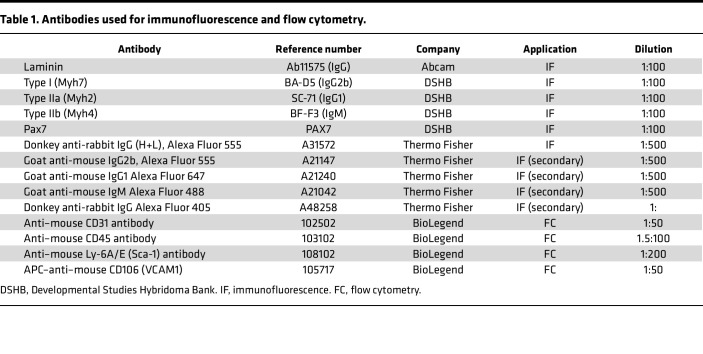
Antibodies used for immunofluorescence and flow cytometry.

## Version 1 03/23/2026

Electronic publication

## References

[1] Tidball JG (2017). Regulation of muscle growth and regeneration by the immune system. Nat Rev Immunol.

[2] Kuang S (2008). Niche regulation of muscle satellite cell self-renewal and differentiation. Cell Stem Cell.

[3] Dumont NA (2015). Intrinsic and extrinsic mechanisms regulating satellite cell function. Development.

[4] Lee JW (2014). Thyroid hormone signaling in muscle development, repair and metabolism. J Endocrinol Diabetes Obes.

[5] Salvatore D (2014). Thyroid hormones and skeletal muscle—new insights and potential implications. Nat Rev Endocrinol.

[6] Muscat GE (1994). Activation of myoD gene transcription by 3,5,3′-triiodo-L-thyronine: a direct role for the thyroid hormone and retinoid X receptors. Nucleic Acids Res.

[7] Brent GA (2012). Mechanisms of thyroid hormone action. J Clin Invest.

[8] Milanesi A (2017). Thyroid hormone receptor alpha is essential to maintain the satellite cell niche during skeletal muscle injury and sarcopenia of aging. Thyroid.

[9] Milanesi A (2016). Thyroid hormone receptor α plays an essential role in male skeletal muscle myoblast proliferation, differentiation, and response to injury. Endocrinology.

[10] Dentice M (2010). The FoxO3/type 2 deiodinase pathway is required for normal mouse myogenesis and muscle regeneration. J Clin Invest.

[11] Dentice M (2013). Type 3 deiodinase and solid tumors: an intriguing pair. Expert Opin Ther Targets.

[12] Sindoni A (2016). Hypothyroid myopathy: a peculiar clinical presentation of thyroid failure. Review of the literature. Rev Endocr Metab Disord.

[13] Oprescu SN (2020). Temporal dynamics and heterogeneity of cell populations during skeletal muscle regeneration. iScience.

[14] Giordani L (2019). High-dimensional single-cell cartography reveals novel skeletal muscle-resident cell populations. Mol Cell.

[15] De Micheli AJ (2020). Single-cell analysis of the muscle stem cell hierarchy identifies heterotypic communication signals involved in skeletal muscle regeneration. Cell Rep.

[16] McKellar DW (2021). Large-scale integration of single-cell transcriptomic data captures transitional progenitor states in mouse skeletal muscle regeneration. Commun Biol.

[17] Simonides WS, van Hardeveld C (2008). Thyroid hormone as a determinant of metabolic and contractile phenotype of skeletal muscle. Thyroid.

[18] Cunningham JT (2007). mTOR controls mitochondrial oxidative function through a YY1-PGC-1alpha transcriptional complex. Nature.

[19] Abe T (2013). Visualization of cell cycle in mouse embryos with Fucci2 reporter directed by Rosa26 promoter. Development.

[20] Mort RL (2014). Fucci2a: a bicistronic cell cycle reporter that allows Cre mediated tissue specific expression in mice. Cell Cycle.

[21] Liu L (2015). Isolation of skeletal muscle stem cells by fluorescence-activated cell sorting. Nat Protoc.

[22] Molina T (2021). Fibro-adipogenic progenitors in skeletal muscle homeostasis, regeneration and diseases. Open Biol.

[23] Muller-Dott S (2023). Expanding the coverage of regulons from high-confidence prior knowledge for accurate estimation of transcription factor activities. Nucleic Acids Res.

[24] Alvarez MJ (2016). Functional characterization of somatic mutations in cancer using network-based inference of protein activity. Nat Genet.

[25] Weitzel JM (2001). Two thyroid hormone-mediated gene expression patterns in vivo identified by cDNA expression arrays in rat. Nucleic Acids Res.

[26] Kress E (2009). Thyroid hormones and the control of cell proliferation or cell differentiation: paradox or duality?. Mol Cell Endocrinol.

[27] Mackey AL (2017). Human skeletal muscle fibroblasts stimulate in vitro myogenesis and in vivo muscle regeneration. J Physiol.

[28] Aguiari P (2021). Persistent COUP-TFII expression underlies the myopathy and impaired muscle regeneration observed in resistance to thyroid hormone-alpha. Sci Rep.

[29] Dentice M, Salvatore D (2011). Deiodinases: the balance of thyroid hormone: local impact of thyroid hormone inactivation. J Endocrinol.

[30] Dentice M (2014). Intracellular inactivation of thyroid hormone is a survival mechanism for muscle stem cell proliferation and lineage progression. Cell Metab.

[31] Almada AE (2021). FOS licenses early events in stem cell activation driving skeletal muscle regeneration. Cell Rep.

[32] Fu X (2015). Stem cell activation in skeletal muscle regeneration. Cell Mol Life Sci.

[33] Safwan-Zaiter H (2022). P16INK4A—more than a senescence marker. Life (Basel).

[34] Mahdavi V (1987). Developmental and hormonal regulation of sarcomeric myosin heavy chain gene family. Circ Res.

[35] Ziemkiewicz N (2021). The role of innate and adaptive immune cells in skeletal muscle regeneration. Int J Mol Sci.

[36] Behmoaras J (2025). The spatial and temporal activation of macrophages during fibrosis. Nat Rev Immunol.

[37] Yin K (2024). FAPs orchestrate homeostasis of muscle physiology and pathophysiology. FASEB J.

[38] Wosczyna MN (2019). Mesenchymal stromal cells are required for regeneration and homeostatic maintenance of skeletal muscle. Cell Rep.

[39] Turowska O (2007). Overexpression of E2F1 in clear cell renal cell carcinoma: a potential impact of erroneous regulation by thyroid hormone nuclear receptors. Thyroid.

[40] Wulf A (2008). T3-mediated expression of PGC-1alpha via a far upstream located thyroid hormone response element. Mol Cell Endocrinol.

[41] Freitas BC (2010). Paracrine signaling by glial cell-derived triiodothyronine activates neuronal gene expression in the rodent brain and human cells. J Clin Invest.

[42] Giolito MV, Plateroti M (2022). Thyroid hormone signaling in the intestinal stem cells and their niche. Cell Mol Life Sci.

[43] Ribeiro MO (2010). Expression of uncoupling protein 1 in mouse brown adipose tissue is thyroid hormone receptor-beta isoform specific and required for adaptive thermogenesis. Endocrinology.

[44] Schiaffino S (1989). Three myosin heavy chain isoforms in type 2 skeletal muscle fibres. J Muscle Res Cell Motil.

[45] Schindelin J (2012). Fiji: an open-source platform for biological-image analysis. Nat Methods.

[46] Stringer C (2021). Cellpose: a generalist algorithm for cellular segmentation. Nat Methods.

[47] Bankhead P (2017). QuPath: open source software for digital pathology image analysis. Sci Rep.

[48] Badia IMP (2022). decoupleR: ensemble of computational methods to infer biological activities from omics data. Bioinform Adv.

[49] Holland CH (2020). Robustness and applicability of transcription factor and pathway analysis tools on single-cell RNA-seq data. Genome Biol.

[50] Mundo AI (2022). Generalized additive models to analyze nonlinear trends in biomedical longitudinal data using R: beyond repeated measures ANOVA and linear mixed models. Stat Med.

[51] Van den Berge K (2020). Trajectory-based differential expression analysis for single-cell sequencing data. Nat Commun.

[52] Patterson-Cross RB (2021). Selecting single cell clustering parameter values using subsampling-based robustness metrics. BMC Bioinformatics.

[53] Turei D (2016). OmniPath: guidelines and gateway for literature-curated signaling pathway resources. Nat Methods.

[54] Dimitrov D (2022). Comparison of methods and resources for cell-cell communication inference from single-cell RNA-Seq data. Nat Commun.

