# Supplementary material for: Hypothyroidism impairs skeletal muscle regeneration after injury by altering myogenic and nonmyogenic pathways
Source: JCI Insight. 2026 Mar 23;11(6):e197761. doi: 10.1172/jci.insight.197761 (PMC13043096; doi:10.1172/jci.insight.197761)
Supplement: Supplemental data [file jciinsight-11-197761-s176.pdf]

Supplementary Material

Hypothyroidism Reduces Impairs Skeletal Muscle Regeneration After Injury by Altering Myogenic and Non-Myogenic Pathways

Paola Aguiari[1,2], Valentina Villani[1], Yan-Yun Liu[2], Gianni Carraro[3,4], Gregory A Brent[2], *Laura Perin[1,4], *Anna Milanesi[2]

[1] GOFARR Laboratory, Children’s Hospital Los Angeles, Division of Urology, Saban Research Institute, Los Angeles, CA, USA

[2] Department of Medicine, David Geffen School of Medicine at UCLA - VA Greater Los Angeles Healthcare System, Los Angeles, CA, USA.

[3] Children's Hospital Los Angeles, Division of Pediatric Surgery, The Saban Research Institute, Los Angeles, CA, USA.

[4] Keck School of Medicine, University of Southern California, Los Angeles, CA, USA

Supplementary Figure 1

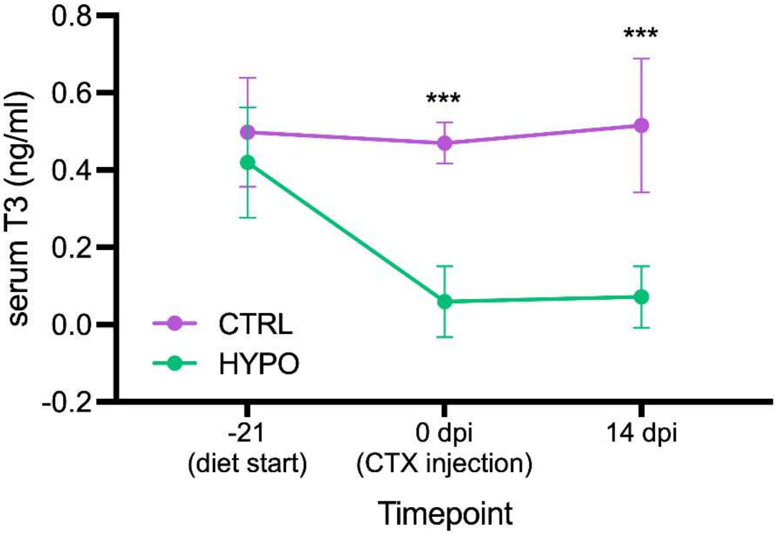

Serum T3 levels in CTRL and hypothyroid mice.

Serum triiodothyronine (T3) concentrations (ng/ml) were measured at day - 21 (start of hypothyroid diet), day 0 (CTX injection), and day 14 (post-injury). Lines represent individual mice; bold lines indicate group means ± SEM. CTRL mice maintained stable T3 levels, whereas hypothyroid mice showed a marked reduction by day 0 that persisted through day 14.

Supplementary Figure 2

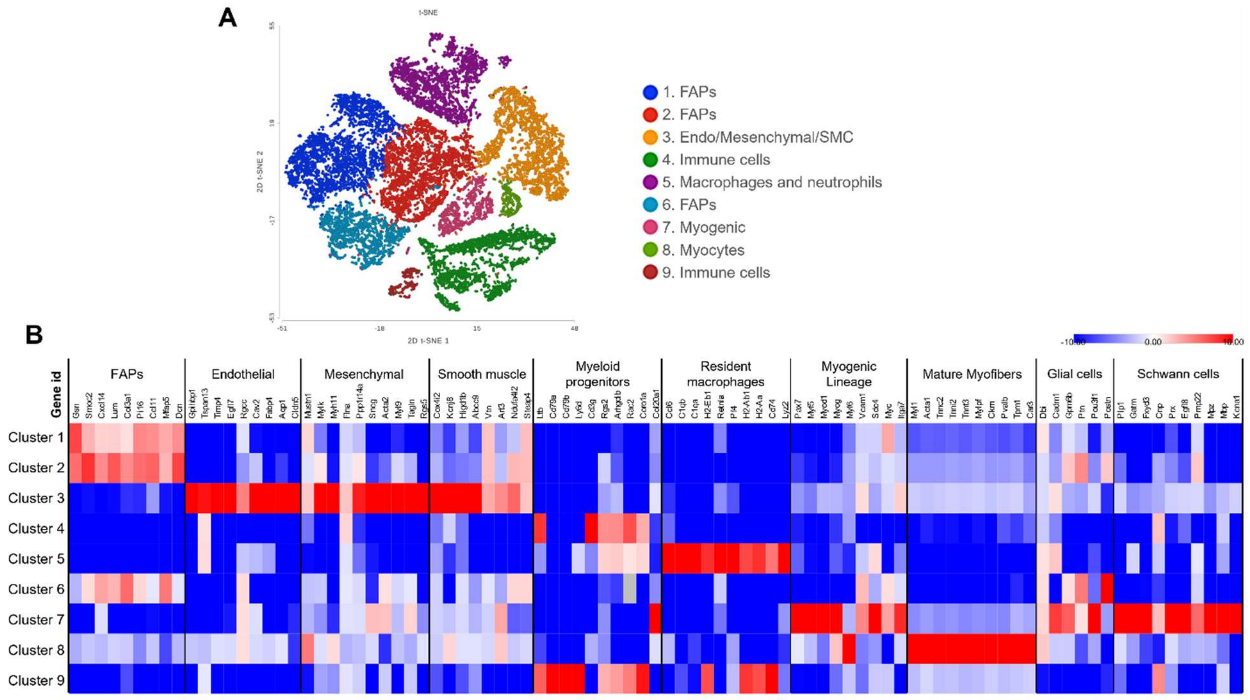

t-SNE visualization of integrated single-cell transcriptomes from skeletal muscle tissues reveals major cell populations. (A) t-SNE projection of 22,409 single cells after graph-based clustering, color-coded by cluster identity. **(B)** Heatmap showing cluster identities assigned based on expression of canonical markers for known skeletal muscle-resident populations. Identified populations include fibro/adipogenic progenitors (FAPs; clusters 1, 2, 6), endothelial/mesenchymal/smooth muscle cells (cluster 3), immune cells (clusters 4, 9), macrophages and neutrophils (cluster 5), myogenic progenitors (cluster 7), and myocytes (cluster 8). In cluster number 7 (myogenic progenitors), we identified expression of Glial cells and Schwann cells markers. Legend: (FAPs) Fibro/Adipogenic Progenitors.

Supplementary Figure 3

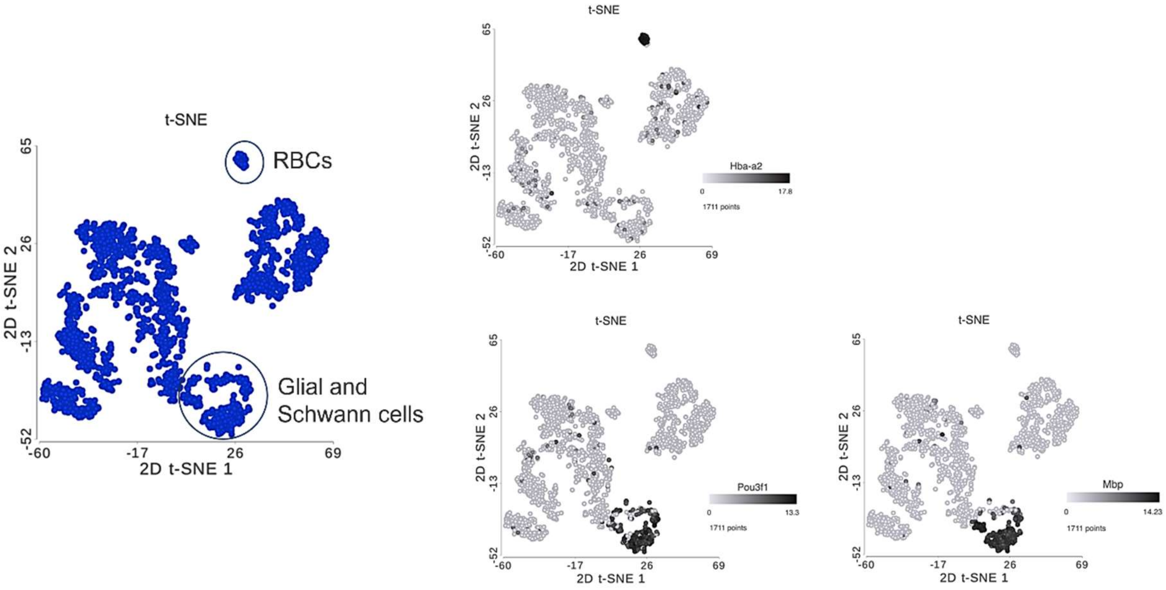

Identification and removal of non-myogenic cell populations from the integrated dataset. t-SNE plots showing the process of filtering out non-myogenic cells from the integrated scRNA-seq dataset. Red blood cells (RBCs) were identified based on Hba-a2 expression (top right panel), and glial/Schwann cells were identified based on the expression of *Pou3f1* and *Mbp* (middle and bottom right panels). Both groups were manually selected (lasso tool) and filtered out.

Supplementary Figure 4

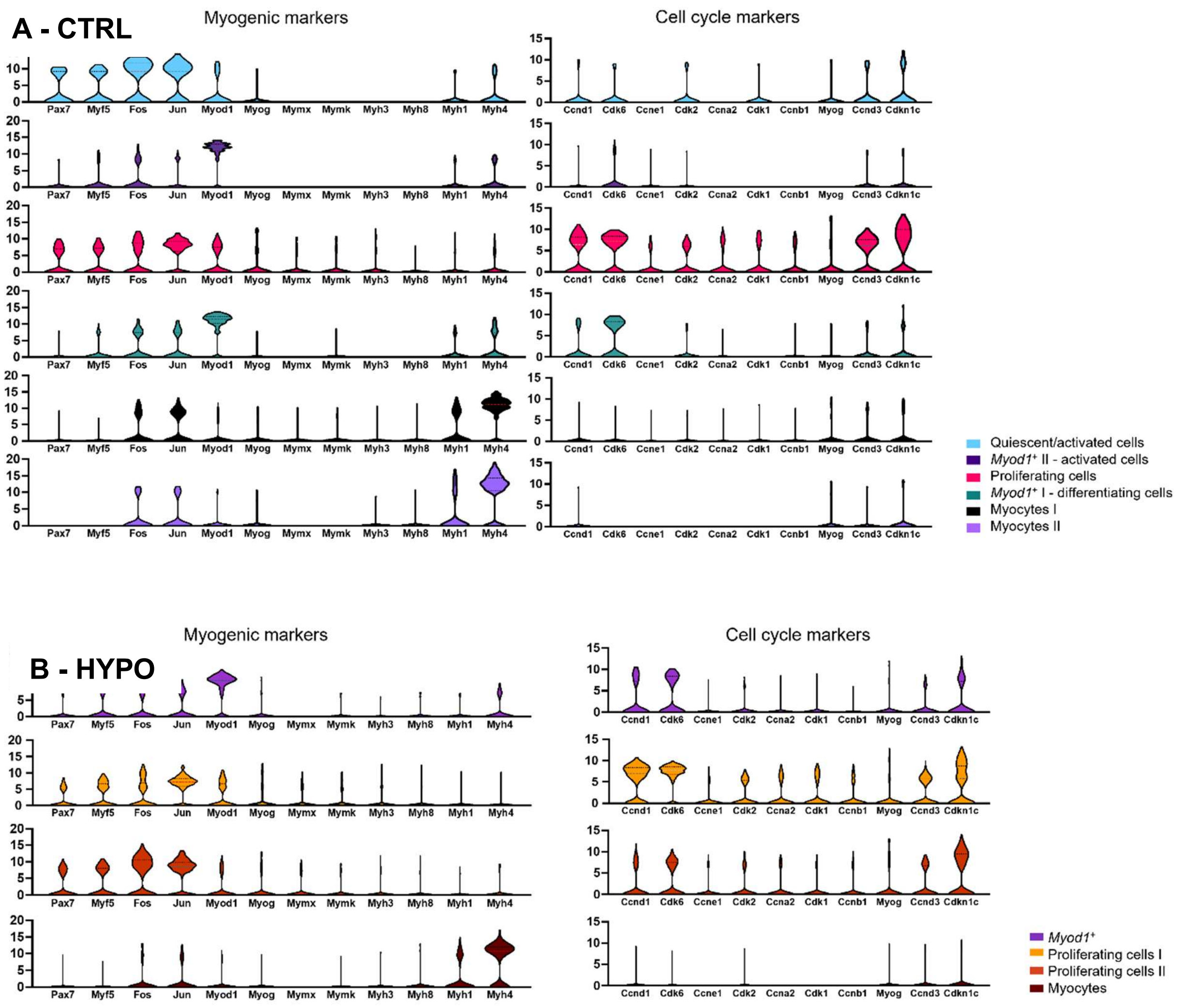

Separated cluster analysis of Control and Hypothyroid conditions reveals transcriptional signatures of muscle stem/progenitor subpopulations. Violin plots showing expression of key myogenic transcription factors (*Pax7*, *Myf5*, *Myod1*, *Myog*), immediate early genes (*Fos*, *Jun*), and cell cycle regulators (e.g., *Ccnd1*, *Cdk1*, Cdk2) across transcriptionally defined clusters from scRNA-seq of control (A - CTRL) and Hypothyroid (B - HYPO) muscle mononuclear cells. In CTRL, six subclusters were identified and annotated as: Quiescent/activated cells (light blue), *Myod1*$^{+}$ II – activated cells (purple), Proliferating cells (pink), *Myod1*$^{+}$ I -differentiating cells (teal), Myocytes I (black), and Myocytes II (purple). In HYPO, four clusters were identified and annotated

as: *Myod1*$^+$ (purple), Proliferating cells I (yellow), Proliferating cells II (orange), and Myocytes (brown). Expression patterns highlight distinct transcriptional programs and altered cell state distributions between conditions. Axes represent normalized expression levels.

Supplementary Figure 5

Myocites I	Myocites II
Citric acid cycle (TCA)	Rho GTPase Signaling Pathways
TCA cycle and respiratory electron transport	RHOBTB GTPase cycle
Respiratory electron transport, ATP synthesis	Cell Cycle
Respiratory electron transport	Translation initiation complex formation
Mitochondrial translation	HSF1 activation
Mitochondrial fatty acid beta-oxidation	rRNA processing (nucleos & cytosol)
mTORC1-mediated signaling	VEGF-VEGFR2 Pathway
MTOR signaling	
Cellular response to stress	

GO Term analysis of Myocite I versus Myocites II (Myocites I) and Myocytes II vs Myocites I (Myocytes II). FDR <0.05.

Supplementary Figure 6

Proliferating I	Proliferating II
MET activates RAP1 and RAC1	Microtubule-dependent trafficking of connexons from Golgi to the plasma membrane
MTOR signaling	Apoptosis induced DNA fragmentation
rRNA processing	Complex I biogenesis
Cellular response to stress	Mitochondrial biogenesis
Cellular senescence	Crosslinking of collagen fibrils
RAF/MAP kinase cascade	RHO GTPase activate IQGAPs
Signaling by interleukins	Recycling pathway of L1
	Selective autophagy
	Signaling by hedgehog
	Cellular response to stress

GO Term analysis of Proliferative I versus Proliferative II (Proliferative I) and Proliferative II vs Proliferative I (Proliferative II). FDR <0.05.

Supplementary Figure 7

CTRL vs Hypo 5 dpi	CTRL vs Hypo 14 dpi
Striate muscle cell differentiation	MET promotes cell mobility
Cell differentiation	Keratan sulfate biosynthesis
Cell activation	Non-Integrin membrane-ECM interactions
Muscle structure development	Elastic fiber formation
Sarcomere organization	Collagen biosynthesis and formation
Muscle contraction	Integrin cell surfase interactions
Muscle adaptation	Assembly of collagen fibrils and other multimeric structure

Gene Ontology (GO) term analysis comparing control and hypothyroid myogenic cells 5- and 14-days post-injury (dpi). FDR <0.05.

Supplementary Figure 8

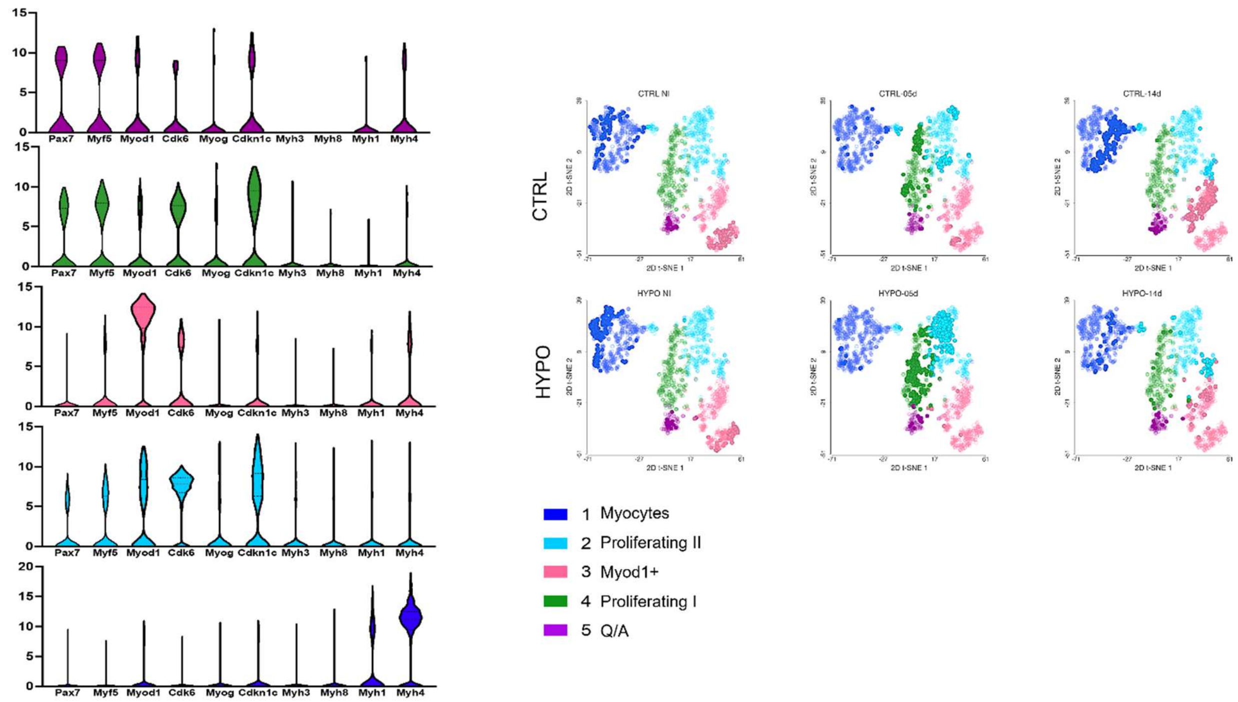

Classification of myogenic clusters and dynamic changes during regeneration in Control and Hypothyroid conditions. (Left) Violin plots of key myogenic and cell cycle markers (e.g., *Pax7*, *Myf5*, *Myod1*, *Cdk6*, *Cdkn1c*, *Myh1*) across five transcriptionally defined cell clusters. Clusters were annotated as: (1) Myocytes (dark blue), (2) Proliferating cells – exiting cycle (light blue), (3) *Myod1*$^{+}$ cells (pink), (4) Proliferating cells – actively cycling (green), and (5) Quiescent/activated (Q/A) cells (purple). (Right) t-SNE plots showing the distribution and abundance of the five clusters in control (CTRL) and hypothyroid (HYPO) muscle samples at three time points during regeneration: non-injured (NI), 5 days post-injury (05d), and 14 days post-injury (14d). Colors correspond to clusters as defined in the violin plots. HYPO samples display altered proportions and regenerative trajectories of myogenic subpopulations compared to CTRL across time points.

Supplementary Figure 9

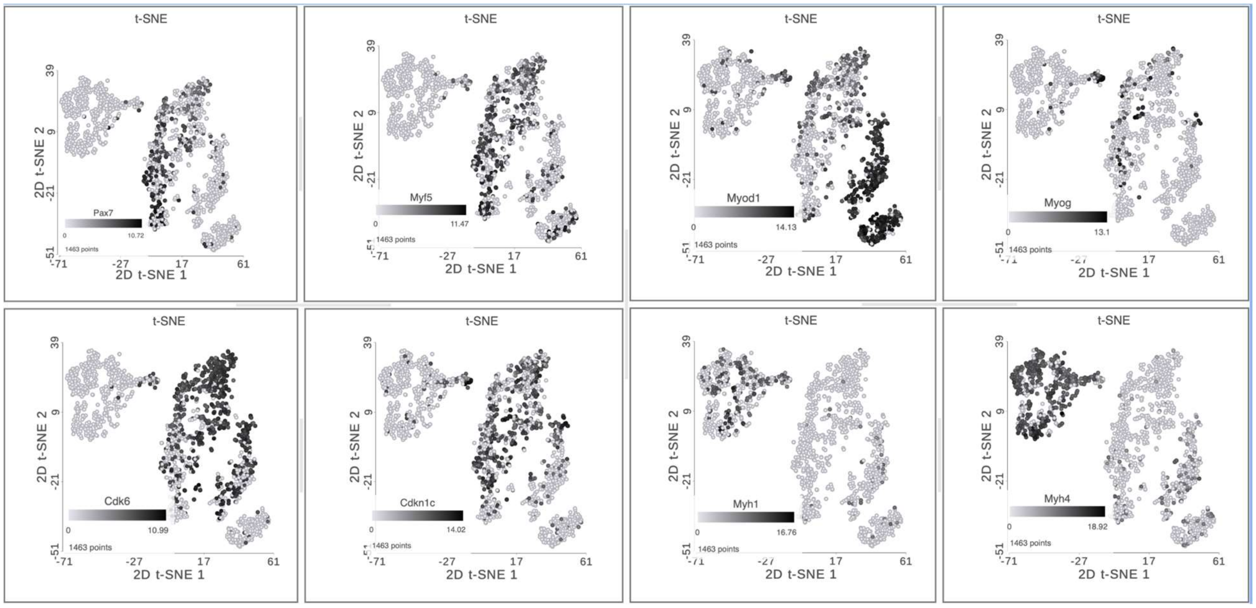

t-SNE feature plots of myogenic marker gene expression in integrated single-cell dataset. t-SNE projections displaying expression levels of canonical myogenic markers across integrated single-cell transcriptomes from control and hypothyroid (HYPO) muscle samples. Each dot represents a single cell (total n = 1463), with grayscale intensity corresponding to normalized expression of the indicated gene. Genes include: *Pax7* (quiescent/activated satellite cells), *Myf5* (early myogenic commitment), *Myod1* (activated/proliferating myoblasts), *Myog* (differentiating myoblasts), *Cdk6* and *Cdkn1c* (cell cycle regulators), *Myh1* and *Myh4* (markers of terminally differentiated myocytes).

Supplementary Figure 10

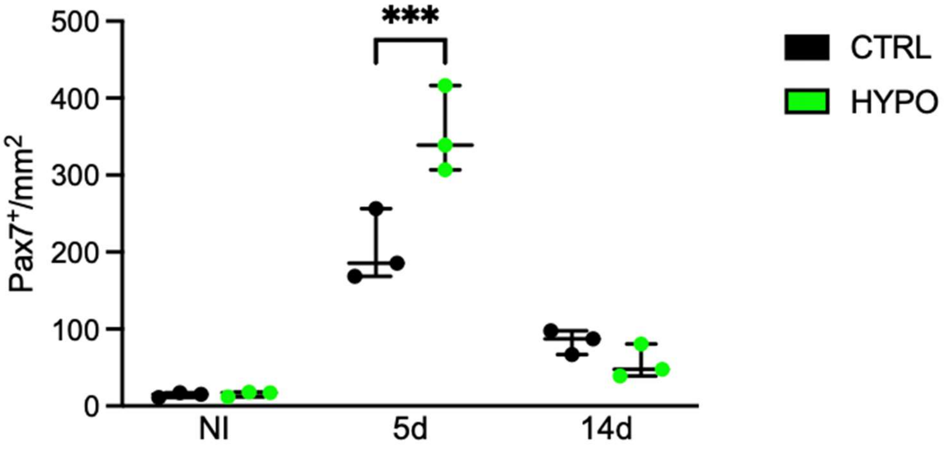

Pax7+ satellite cell abundance is transiently elevated in hypothyroid muscle following injury. Graph showing the quantification of PAX7 cells per mm^2 in tibialis anterior muscle sections from control (CTRL, black) and hypothyroid (HYPO, green) mice under non-injured (NI), 5 days post-injury (5d), and 14 days post-injury (14d) conditions, revealing a significant increase in PAX7 cells in hypothyroid muscle compared to controls at 5 days post-injury, whereas no differences were observed at baseline (NI) or at 14 days. Each point represents an individual mouse; horizontal lines indicate mean ± SD. ***p < 0.001 (unpaired t-test).

Supplementary Figure 11

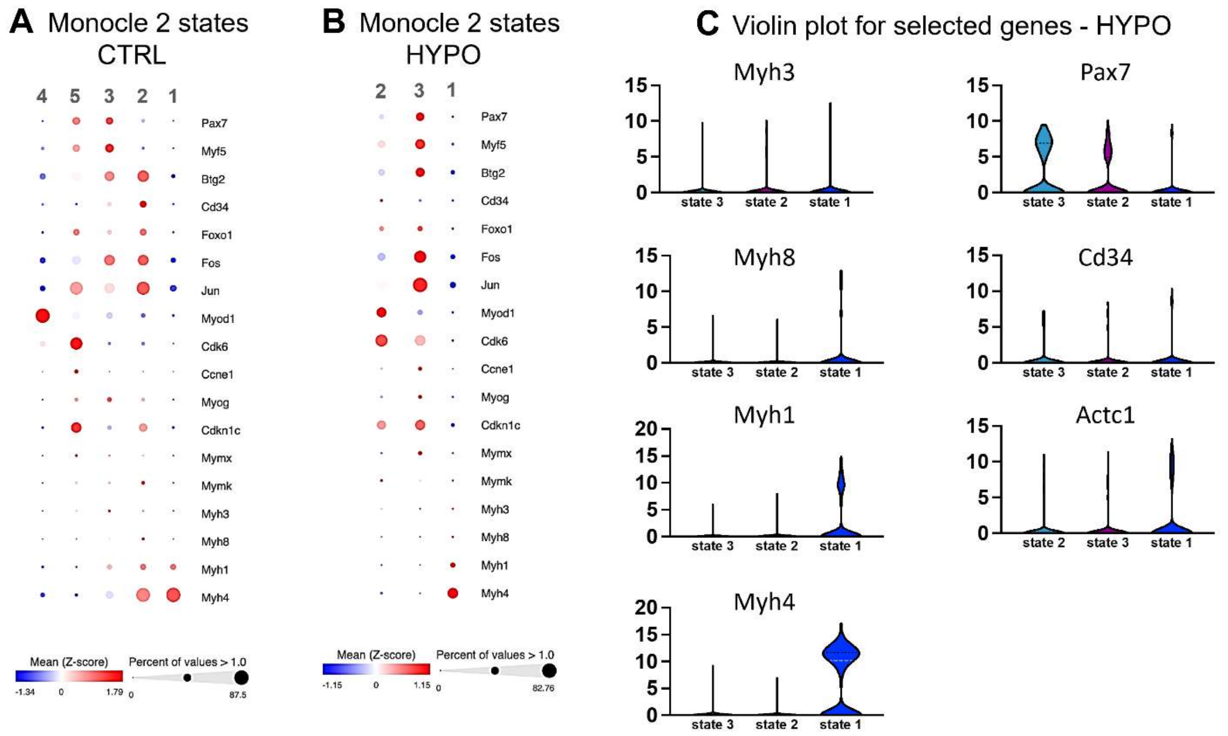

Expression patterns of myogenic regulatory genes. (A, B) Dot plots showing the mean expression (Z-score) and percentage of cells expressing each indicated gene (values >1.0) in different clusters for control **(A)** and hypothyroid **(B)** samples. Color intensity reflects the average scaled expression, while dot size indicates the proportion of cells with detectable expression. Key myogenic markers shown include stem/progenitor markers (*Pax7*, *Myf5*, *Cd34*), early activation markers (*Fos*, *Jun*, *Btg2*), cell cycle regulators (*Cdk6*, *Ccne1*, *Cdkn1c*), and differentiation/fusion markers (*Myod1*, *Myog*, *Mymx*, *Mymk*, *Myh1*, *Myh3*, *Myh4*, *Myh8*). **(C)** Violin plots illustrating the distribution of expression levels for selected genes across clusters, highlighting the dynamic regulation of transcription factors and structural genes during myogenic progression in the hypothyroid sample (HYPO). Each violin plot represents the full distribution of normalized gene expression values within each cluster.

Supplementary Figure 12

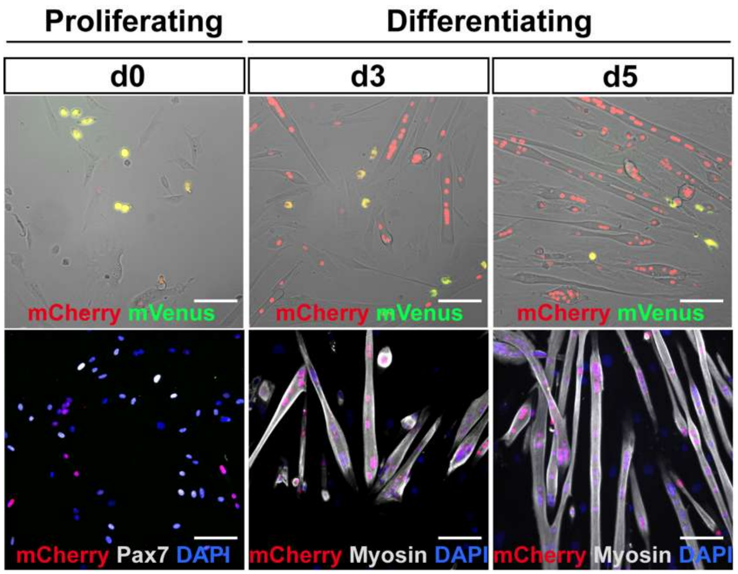

In vitro differentiation of MuSCs from PAX7-FUCCI mice. Representative fluorescence and Bright Field images of proliferating and differentiating primary myoblasts from *Pax7*-FUCCI mice isolated from uninjured TAMs expressing the FUCCI system fluorophores mCherry/G1 (red) andmVenus/G2/M (green) (upper panels) and expressing PAX7 (grey), Myosin (grey) and mCherry (red) (lower panels, nuclei are in blue/DAPI). Bar: 50 μm.

Supplementary Figure 13

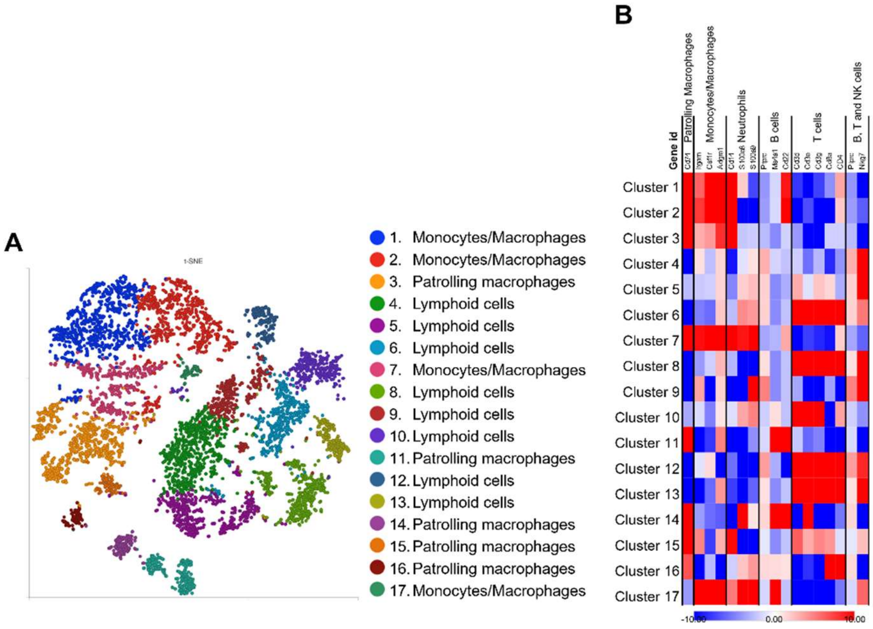

C

%	CTRL			HYPO			
cluster	NI	5d	14d	NI	5d	14d	tot
11	100.0	100.0	100.0	100.0	99.1	100.0	99.6
14	100.0	97.7		100.0	100.0		97.8
15		98.0	100.0		100.0		98.1
16		76.9	100.0	100.0	88.0		80.2

scRNA-seq reveals diverse immune and stromal populations in injured muscle. t-SNE projection **(A)** of 6,753 single cells isolated from muscle tissue across multiple experimental conditions, colored by graph-based clustering (resolution = 1). **(B)** Heatmap showing cluster identities assigned based on specific immune markers expression. Cell types identified include monocytes/macrophages (with neutrophils, cluster 1, 2, 7,17), patrolling macrophages (clusters 3, 11, 14, 15, 16) and lymphoid cells (B, T and NK cells, clusters 4, 5, 6, 8, 9, 10, 12, 13). (**C**) Percentage of $Cd74^{+}$ cells in selected clusters: despite the upregulation of lymphoid markers, clusters 11, 14, 15, and 16 presented high percentages of cells positive for Cd74, a marker of patrolling macrophages. Legend: (CTRL) control; (HYPO) hypothyroid; (NI) before injury; (5d) 5 days, (14d) 14 days.

Supplementary Figure 14

A

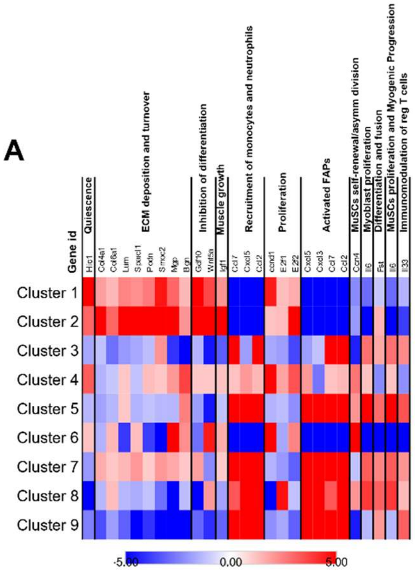

B

	Fold Change			
**cluster**	**NI**	**5d**	**14d**	**Description**
I	**1.1**	**-1.9**	**-1.5**	Homeostatic 1
II	**1.7**	**-1.5**	**-2.3**	Homeostatic 2
III	**-1.2**	**-1.1**	**+4.2**	Recruitment of monocytes and neutrophils
IV	0.0	**-1.1**	+12.5	Proliferating
V	0.0	**+1.3**	+6.7	Activated
VI	/	-1.9	**-3.0**	Promoting MuSCs self-renewal and asymmetric division
VII	0.0	+1.2	**+2.2**	Promoting proliferation, differentiation and fusion
VIII	0.0	+16.2	**-1.1**	Recruitment of monocytes and neutrophils, inhibiting differentiation, promoting proliferation
IX	-2.0	-3.1	**-1.2**	Immunomodulation of regulatory T cells

scRNA-seq shows dynamic changes in FAPs populations in injured muscle. (A) Heatmap showing the scaled expression (Z-score) of key marker genes across 9 fibro-adipogenic progenitor (FAP) clusters identified by scRNA-seq analysis. Each row represents a distinct FAP cluster, while columns indicate representative genes grouped by functional annotation, including quiescence (e.g., *Htra1, Ccl11*), ECM deposition and turnover (*Col1a1, Col3a1*), inhibition of differentiation (*Sfrp4, Dpp4*), tissue growth (*Bmp4*), immune crosstalk (*Ccl2, Ccl7, Ccl8*), proliferation (*Ccnb1, E2f1*), activated FAPs (*Cd74, Cd83*), muscle stem cell (MuSC) regulation (*Gas1, Ccnd2, Igfbp6*), myogenic progression (*Fgfr2, Fst*), and immunomodulation of regulatory T cells (*Il33*). Red indicates higher expression, and blue indicates lower expression. (**B**) Table showing the change in relative abundance of cells in FAP clusters presented as fold change: number in bold represent significant differences (fold change from clusters with a number of cells higher than 30 for both CTRL and HYPO samples); numbers in grey represent clusters with a

number of cells lower than 30 for both CTRL and HYPO samples. Legend: (NI) before injury; (5d) 5 days, (14d) 14 days.

Supplementary Figure 15

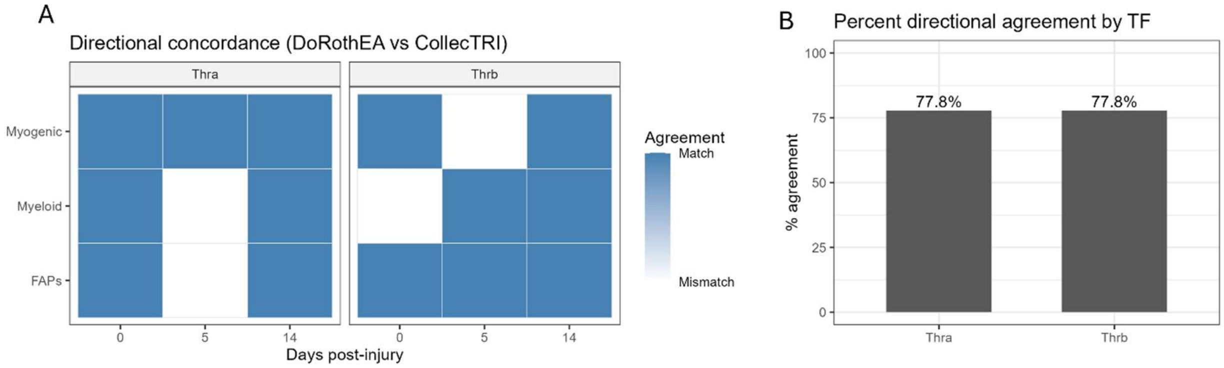

Validation of CollectTRI-inferred THR activity with DoRothEA regulons. (A) Heatmap of directional concordance showing whether the sign of the HYPO vs. CTRL effect size for *Thra* and *Thrb* activity matched between CollectTRI and DoRothEA regulon analyses across cell types (FAPs, Myeloid, Myogenic) and time points (0, 5, and 14 days post-injury). Blue squares indicate matching direction of effect; white squares indicate a mismatch. (**B)** Bar plot summarizing the overall percent of cell-type/time comparisons in which the direction of the effect agreed between frameworks. Approximately 78 % directional agreement was observed for both *Thra* and *Thrb*.

Supplementary Figure 16

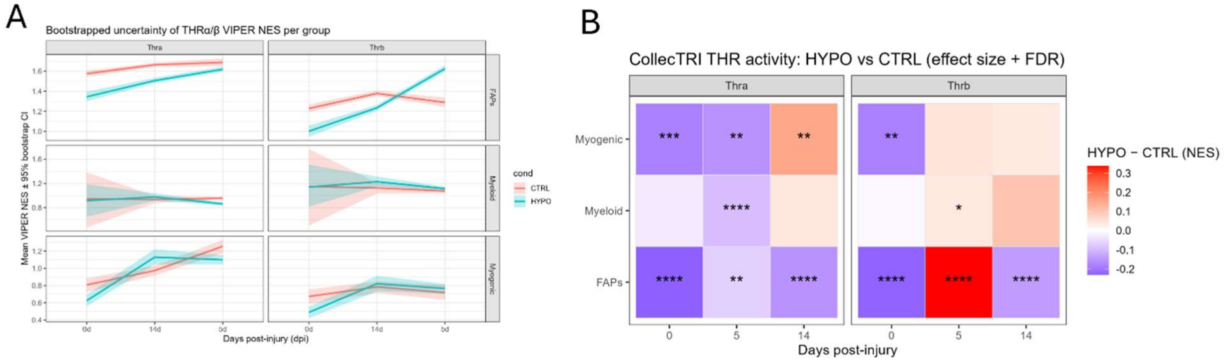

Dynamic changes of *Thra/b* activity during muscle regeneration under hypothyroidism. (A) Bootstrapped modeling of *Thra/b* activity reveals temporal divergence under hypothyroidism. To assess the robustness of inferred transcriptional activity patterns in the absence of biological replication, we performed 1,000-fold bootstrapping of per-cell VIPER NES values within each sample. The resulting shaded confidence ribbons capture the variability expected from cellular and technical heterogeneity within each mouse rather than between animals. Despite this limitation, consistent temporal patterns emerged across FAPs and myogenic populations. In FAPs, *Thra* activity in hypothyroid mice (HYPO) rose gradually and approached control levels by 14 dpi, while *Thrb* showed a sustained increase beyond control, suggesting compensatory signaling late in regeneration. In Myogenic cells, *Thra* activity transiently peaked early (5 dpi) in controls but remained blunted and less dynamic in hypothyroid muscle, consistent with delayed activation and reduced transcriptional cycling. **(B) Differential *Thra/b* transcriptional activity in control versus hypothyroid muscle regeneration.** Heatmap showing HYPO-CTRL differences in VIPER-inferred normalized enrichment scores (NES) for ***Thra*** (left) and ***Thrb*** (right) regulons across major cell populations (Myogenic, Myeloid, and FAPs) at 0, 5,and 14 days post-injury (dpi). Color intensity represents the magnitude and direction of change (red = higher ***Thr*** activity in HYPO; blue = lower activity in HYPO). Asterisks denote FDR-adjusted Wilcoxon p-values (p < 0.05 = * < 0.01 = **, < 0.001 = ***, < 0.0001 = ****).

Supplementary Figure 17

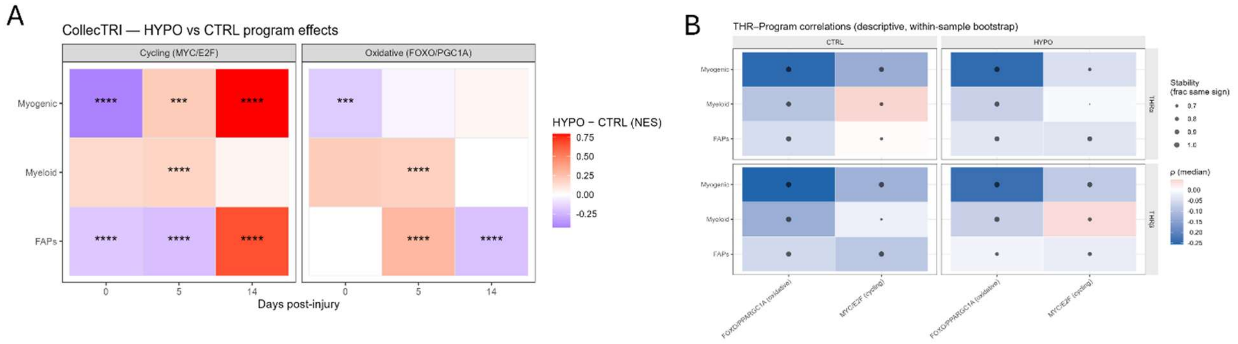

Hypothyroidism disrupts THR-driven coordination of proliferative and metabolic programs. (A) CollectTRI-VIPER program effect sizes under hypothyroidism. Heatmaps show HYPO- CTRL differences (delta NES) for the *Myc*/*E2f* and *Foxo*/*Ppargc1a*transcriptional programs across FAP, myogenic, and myeloid populations at 0, 5, and 14 days post-injury. Color scale represents effect magnitude (red = higher in HYPO, blue = lower in HYPO). Asterisks indicate FDR-adjusted Wilcoxon p-values (* < 0.05, ** < 0.01, *** < 0.001, **** < 0.0001). Hypothyroidism transiently enhanced oxidative signatures early (5 dpi) while increasing proliferative *Myc*/*E2f* activity at 14 dpi, indicating phase-shifted transcriptional activation relative to euthyroid regeneration. **(B) Coupling between *Thra/b* activity and regenerative transcriptional programs.** Bootstrapped, within sample correlations between VIPER-inferred thyroid hormone receptor (*Thra, Thrb*) activities and CollectTRI-derived transcriptional programs. Each tile shows the median Spearman across single cells of the indicated type under CTRL or HYPO conditions (3 mice pooled per group). Color intensity denotes correlation direction and magnitude (blue = negative, red = positive); dot size reflects stability (fraction of 1,000 bootstraps retaining the same sign). In myogenic cells, *Thra/b* activities negatively correlated with *Foxo*/*Ppargc1a* and positively with *Myc*/*E2f* programs, supporting a THR-driven coordination of proliferative and metabolic states. These relationships weakened under hypothyroidism and were minimal in FAPs or myeloid populations. Because replicates were pooled, the analysis is descriptive, capturing within-sample variability.

Supplementary Figure 18

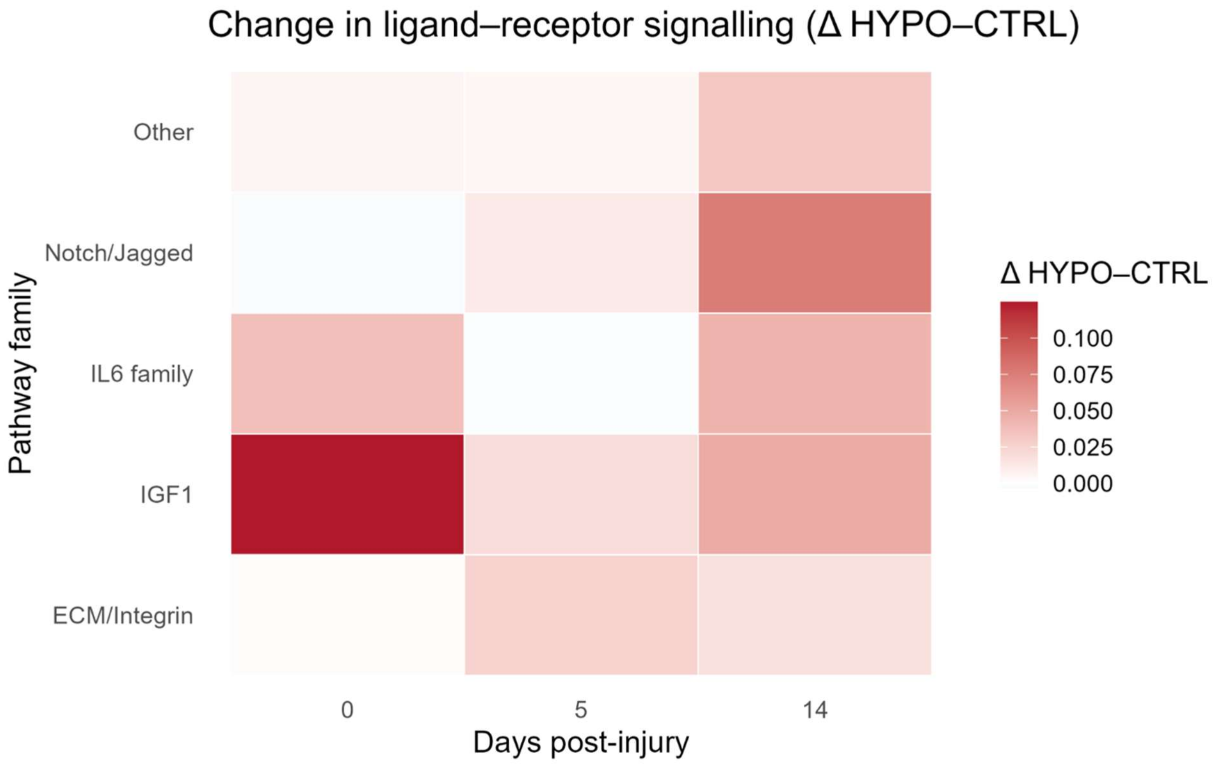

Differential pathway activity in ligand–receptor networks under hypothyroidism. Heatmap summarizing pathway-level changes in LR activity across timepoints. Each row corresponds to a major pathway family (ECM/Integrin, IGF1, IL-6 family cytokines, Notch/Jagged, and Other), and color intensity represents the change in mean LR interaction scores (HYPO - CTRL). Positive values (red shades) indicate stronger signaling under hypothyroid conditions, while lighter colors indicate minimal or absent differences.

Supplementary Figure 19

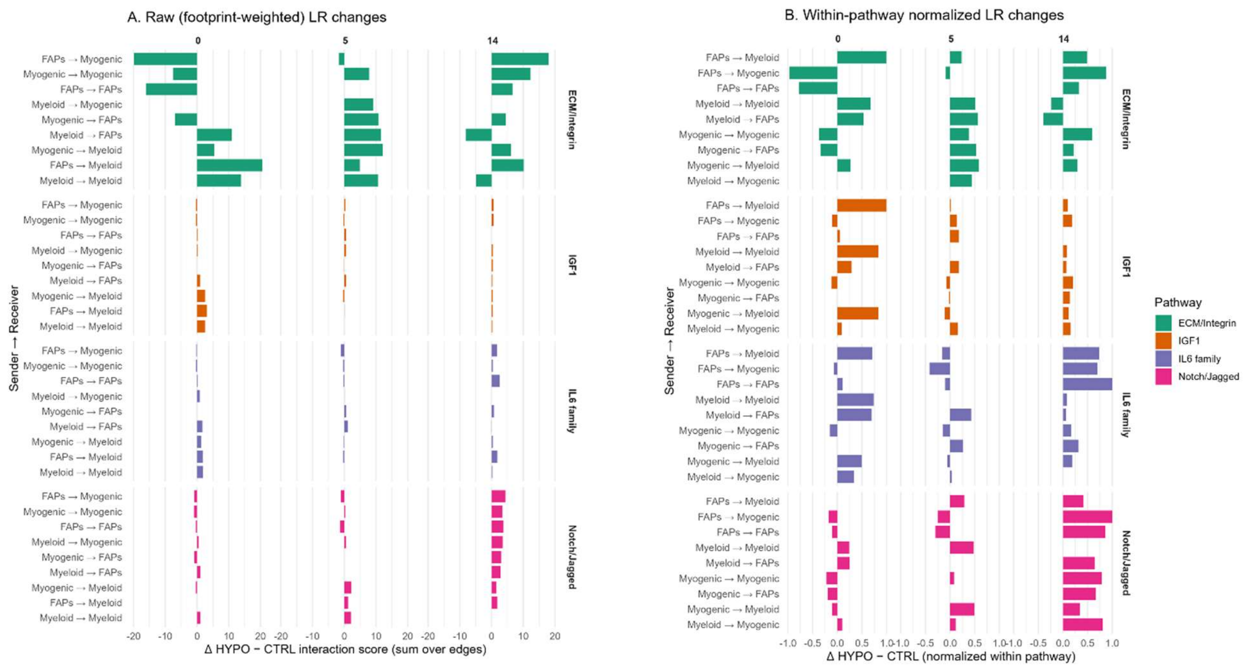

Raw ligand-receptor interaction changes across selected pathways. (A) Barplots showing footprint-weighted, unnormalized changes in LR interaction scores (HYPO - CTRL) for selected pathways (ECM/Integrin, IGF1, IL-6 family, Notch/Jagged). Each panel corresponds to a pathway, stratified by sender to receiver direction and regeneration stage (0, 5, 14 dpi). The raw magnitude of changes highlights the absolute dominance of ECM/Integrin signaling, with large positive and negative shifts depending on sender-receiver direction. IGF1 and IL-6 signals appear comparatively weaker in magnitude but show strong temporal specificity (IGF1 early, IL-6 late). (**B**) Barplots representing within-pathway normalized LR interaction scores (HYPO - CTRL, scaled between -1 and +1). Normalization reduces the overwhelming contribution of ECM/Integrin edges, thereby allowing direct visual comparison of temporal dynamics across pathways.

Supplementary Table 1. Relative proportion (shown as percentages of the total number of cells in the sample) and fold change for proliferating cells, *Myod1*$^{+}$ cells, and differentiated myocytes in control and hypothyroid myogenic datasets at different timepoints. FC > 1.5 is in red and FC < -1.5) is in blue. Legend: (CTRL) control; (HYPO) hypothyroid; (NI) before injury; (5d) 5 days, (14d) 14 days.

		NI	5d	14d
**Proliferating cells**	CTRL	0.0	78.6	3.2
	HYPO	9.1	96.0	32.4
	**Fold change**		**1.2**	**10.1**
**Myod1+**	CTRL	40.5	10.4	41.0
	HYPO	24.5	1.8	47.3
	**Fold change**	**-1.7**	**-6.0**	**1.2**
**Myocytes**	CTRL	52.1	7.5	49.4
	HYPO	66.3	2.3	20.3
	**Fold change**	**1.3**	**-3.3**	**-2.4**
